# Numerical Investigation of Hydrogen Leakage Quantification and Dispersion Characteristics in Buried Pipelines

**DOI:** 10.3390/ma18194535

**Published:** 2025-09-29

**Authors:** Yangyang Tian, Jiaxin Zhang, Gaofei Ren, Bo Deng

**Affiliations:** 1 College of Petroleum Engineering, Xi’an Shiyou University, Xi’an 710065, China; zhangjx200017@163.com; 2 Shaanxi Key Laboratory of Advanced Stimulation Technology for Oil & Gas Reservoirs, Xi’an 710065, China; 3 China Petroleum Pipeline Engineering Corporation, Langfang 065000, China; 13061256562@163.com; 4 Tianshi Group Energy Co., Ltd., Panjin 124221, China; tscitg@163.com

**Keywords:** buried pure hydrogen pipeline, leakage dispersion, numerical simulation, influencing factors

## Abstract

As a clean energy carrier, hydrogen is essential for global low-carbon energy transitions due to its unique combination of safe transport properties and energy density. This investigation employs computational fluid dynamics (ANSYS Fluent) to systematically characterize hydrogen dispersion through soil media from buried pipelines. The research reveals three fundamental insights: First, leakage orifices smaller than 2 mm demonstrate restricted hydrogen migration regardless of directional orientation. Second, dispersion patterns remain stable under both low-pressure conditions (below 1 MPa) and minimal thermal gradients, with pipeline temperature variations limited to 63 K and soil fluctuations under 40 K. Third, dispersion intensity increases proportionally with higher leakage pressures (exceeding 1 MPa), greater soil porosity, and larger particle sizes, while inversely correlating with burial depth. The study develops a predictive model through Sequential Quadratic Programming (SQP) optimization, demonstrating exceptional accuracy (mean absolute error below 10%) for modeling continuous hydrogen flow through moderate-porosity soils under medium-to-high pressure conditions with weak inertial effects. These findings provide critical scientific foundations for designing safer hydrogen transmission infrastructure, establishing robust risk quantification frameworks, and developing effective early-warning systems, thereby facilitating the practical implementation of hydrogen energy systems.

## 1. Introduction

Hydrogen energy is recognized as a versatile energy carrier with the potential to be a low-carbon and eco-friendly resource when produced from renewable sources (green hydrogen) or from fossil fuels with carbon capture and storage (blue hydrogen). It is pivotal in establishing clean, secure, and efficient energy systems [1,2,3,4,5]. However, it is important to note that conventional hydrogen production from fossil fuels without emission mitigation (gray hydrogen) does not share these environmental benefits. The large-scale implementation of the hydrogen economy, however, critically depends on solving the challenge of efficient and safe transportation. Pipeline transmission, recognized for its high efficiency and economic viability [6,7,8], is the most promising solution for long-distance hydrogen delivery.

Global hydrogen pipeline infrastructure, originating in the late 1930s in Europe and North America, now exceeds 5000 km, primarily distributed in the United States (52%) and Europe (35%), with the remainder in Asia-Pacific nations [9,10]. This maturation has spurred international design standards like ASME B31.12-2019 [11], IGC Doc 121/14 [12], and CGA G-5.6-2005 [13]. In contrast, China’s long-distance hydrogen pipeline network is still nascent, with plans for ~1800 km of dedicated and blended pipelines, typically designed for diameters of 250~500 mm and pressures below 5 MPa [14,15,16].

A paramount safety concern for hydrogen pipelines is leakage, particularly from buried infrastructure operating under sustained high pressure and cyclic stresses [17,18]. Leaked hydrogen, accumulating in confined spaces, poses severe detonation risks due to its wide explosive range and low ignition energy. Consequently, understanding subsurface hydrogen dispersion is essential for risk assessment and safety management. While significant research has been conducted on leakage from pipelines transporting hydrogen–natural gas blends [19,20,21,22,23] or other hazardous gases [24,25], these studies are insufficient to address the risks specific to pure hydrogen systems due to fundamental differences in physicochemical properties.

A summary of key experimental, numerical, and accident-based research studies on the leakage and dispersion characteristics from buried hydrogen-related pipelines is provided in Table 1. The table compiles investigations into critical parameters such as leakage hole size, pipeline operating pressure, burial depth, and soil properties.

As evidenced by the table, to ground this study in practical engineering contexts, a review of historical hydrogen pipeline incident data was conducted using the Pipeline and Hazardous Materials Safety Administration (PHMSA) database [32]. The search revealed a very limited number of recorded incidents, with only one pertaining to buried pipelines experiencing pinhole leaks. While these real-world cases provide invaluable context and confirm the occurrence of the failure modes studied herein, a direct quantitative benchmarking of the present model against these specific incidents is not feasible. This is due to the critical absence of precise parameters in the incident reports, such as the exact leakage orifice diameter, soil properties at the failure location, and the precise leakage duration. The lack of publicly available, high-fidelity field data further underscores the value of rigorously validated computational models, as presented in this work, for performing risk assessment and consequence analysis where empirical data is scarce.

Two principal modeling frameworks dominate leakage rate analysis: full-bore leakage models and small-orifice leakage models. The former addresses leakage rate estimation under full-bore rupture scenarios requiring complete cross-sectional failure, while the latter focuses on leakage rate estimation through discrete orifices. Contemporary research has developed sophisticated small-orifice models integrating subsurface transport mechanisms with multi-parametric considerations (soil porosity, pressure gradients, and gas composition) to enable precise prediction of natural gas/hydrogen blend dispersion in buried infrastructure. Table 2 summarizes critical parameters governing small-orifice leakage models, including soil porosity, pressure gradients, and gas composition.

Current research on hydrogen leakage behavior exhibits critical limitations in three principal areas: (i) Low-Pressure Limitations: Existing models and experimental studies are predominantly restricted to low-pressure conditions (<0.5 MPa), failing to represent real-world pipeline operating pressures (2–6 MPa), where high-pressure effects such as compressibility and choked flow fundamentally alter leakage dynamics; (ii) Neglect of Soil Interactions: Many studies prioritize aboveground leakage scenarios, overlooking essential soil–gas interactions—including porosity, particle size, and permeability—that dictate subsurface dispersion behavior; and (iii) Oversimplified Leak Characterization: Influential morphological factors such as leak orientation are frequently disregarded [32,33,34,35,36], leading to an incomplete understanding of hazard zone formation and dispersion patterns.

This lack of understanding surrounding high-pressure pure hydrogen leakage and its subsequent migration through soil represents a critical research gap that impedes the development of accurate predictive tools and effective safety protocols for dedicated hydrogen pipeline infrastructure. Nevertheless, recent advances in numerical simulations of gas transport through porous media have improved capabilities for predicting subsurface flow dynamics [1,38]. Additionally, insights derived from research on underground hydrogen storage (UHS) [39,40], which examines long-term hydrogen–soil interactions on a different scale, offer a valuable foundation for further investigation.

To bridge this gap, this study employs a comprehensive numerical framework to investigate the dispersion characteristics of high-pressure (up to 6 MPa) pure hydrogen from leaks in buried pipelines. We systematically quantify the influence of eight key parameters, explicitly extending beyond previous studies by:

(i) Investigating actual transmission-level pressures (0.5~6 MPa).
(ii) Incorporating leak orientation and thermal effects (soil and pipeline temperature).
(iii) Fully integrating key soil properties (porosity, particle size) as core variables.
(iv) Expanding the analysis of burial depth and orifice diameter.

We systematically quantify the influence of eight key parameters, explicitly extending beyond previous studies by incorporating leak orientation and thermal effects (soil and pipeline temperature), in addition to key factors like orifice diameter, pressure, burial depth, and soil properties (porosity and particle size). The primary objective is to identify the dominant factors controlling leakage and dispersion, thereby providing a fundamental basis for developing quantitative risk assessment models and informing the design of monitoring systems for the emerging hydrogen pipeline infrastructure.

## 2. Numerical Model Framework for Buried Hydrogen Pipelines Leakage

Prior to developing the computational model for hydrogen dispersion in buried pipelines, we implement three fundamental assumptions to balance physical fidelity with computational tractability. These simplifications are necessary to establish a foundational understanding of the dispersion mechanics, though they introduce limitations regarding field-scale heterogeneity, as discussed in Section 5.2.

(1) Non-reactive transport: The hydrogen–soil system is modeled as a non-reacting multicomponent mixture, with steady-state leakage conditions maintained at constant orifice pressure. This assumption is justified for the high-flow, advection-dominated leakage scenarios that are the primary focus of this study. Under these conditions, the residence time of hydrogen in the soil is short, and the physical processes of advection and turbulent dispersion are expected to dominate the transport mechanics over potential biogeochemical reactions (e.g., microbial oxidation, adsorption, embrittlement of pipeline steels). Therefore, this assumption allows us to isolate and elucidate the dominant physical dispersion phenomena, providing a conservative (i.e., potentially higher concentration) estimate of gaseous hydrogen distribution in the short term.
(2) Homogeneous media: The soil matrix is treated as an isotropic, homogeneous porous medium saturated with dry air (water content is 0%), transporting pure hydrogen. This simplification is a common starting point in foundational studies [1,21,33] to elucidate first-order effects. We explicitly acknowledge that natural soils are often anisotropic, stratified, and contain varying degrees of moisture. The exclusion of water saturation, while a necessary simplification for this foundational study, is a significant limitation, as groundwater presence could drastically alter the system’s behavior. Moisture content critically reduces effective porosity and gas-phase diffusion coefficients, thereby trapping hydrogen and altering its dispersion pattern. Furthermore, water saturation significantly impacts relative permeability, which governs the flow capacity of the gas phase, potentially creating localized accumulations and preferential flow paths. The choice of dry, homogeneous soil provides a crucial baseline case for understanding the fundamental physics; the critical investigation of moisture effects is prioritized as the most immediate avenue for future research (see Section 5.2).
(3) Small-orifice regime: Leakage scenarios are limited to orifice diameters d_o_ ≤ 20 mm, with viscous resistance coefficient (α) and inertial resistance coefficient (C2) calculated via Equations (1) and (2) to characterize porous media resistance. This focuses the study on the most common pipeline leakage scenarios, excluding large-scale ruptures.

(1) $$\frac{1}{\alpha }=\frac{150}{d_{s}^{2}}\frac{{\left(1-\varphi \right)}^{2}}{\varphi^{3}}$$

(2) $$C_{2}=\frac{3.5}{d_{s}}\frac{\left(1-\varphi \right)}{\varphi^{3}}$$

### 2.1. Governing Equations

The hydrogen leakage and dispersion process is governed by fundamental conservation laws, expressed through the following coupled partial differential equations:

Continuity equation:

(3) $$\frac{𝜕\rho }{𝜕t}+\frac{𝜕(\rho u_{i})}{𝜕x_{i}}=0$$

where *ρ* represents the fluid density (kg/m^3^); *u_i_* denotes the velocity in the *X*, *Y* and Z directions (m/s).

Momentum equation (Navier–Stokes):

(4) $$\rho (\frac{𝜕u_{i}}{𝜕t}+u\nabla u)=-\nabla p+\mu \nabla^{2}u+\rho f$$

where f represents the body force per unit mass (m/s^2^); *u* indicates the velocity (m/s); *μ* denotes the dynamic viscosity (Pa·s).

Energy equation:

(5) $$\frac{𝜕(\rho E)}{𝜕t}+\nabla \left[u_{i}(\rho E+p)\right]=\nabla \left[\left(k_{eff}+\frac{c_{p}\mu_{t}}{P_{rt}}\right)\frac{𝜕T}{𝜕x_{j}}+u_{i}{\left(T_{ij}\right)}_{eff}\right]$$

where *E* represents the total energy of the fluid element (J); $k_{eff}$ indicates the effective thermal conductivity (W/(m·K)); *c_p_* denotes the specific heat at constant pressure (m^2^/(s^2^∙K));$\mu_{t}$ represents the turbulent viscosity (Pa·s); $P_{rt}$ indicates the turbulent Prandtl number (-); *T* denotes the temperature (K); ${\left(T_{ij}\right)}_{eff}$ represents the effective deviatoric stress tensor (Pa).

*k*-*ω* turbulence equation:

*k*-equation:

(6) $$\frac{𝜕}{𝜕t}\left(\rho k\right)+\frac{𝜕}{𝜕x_{i}}\left(\rho ku_{i}\right)=\frac{𝜕}{𝜕x_{j}}\left[\Gamma_{k}\frac{𝜕k}{𝜕x_{j}}\right]+G_{k}-Y_{k}+S_{k}+G_{b}$$

*ω*-equation:

(7) $$\frac{𝜕}{𝜕t}\left(\rho \omega \right)+\frac{𝜕}{𝜕x_{i}}\left(\rho \omega u_{i}\right)=\frac{𝜕}{𝜕x_{j}}\left[\Gamma_{\omega }\frac{𝜕\omega }{𝜕x_{j}}\right]+G_{\omega }-Y_{\omega }+S_{\omega }+G_{\omega b}$$

where *G_k_* denotes the turbulent kinetic energy production due to the mean velocity gradient (m^2^/s^3^); *G_ω_* denotes the generation of ω (s^−2^); Γ*_k_* represent the effective diffusivity of *k* (m^2^/s^3^); Γ*_ω_* represent the effective diffusivity of *ω* (m^2^/s); *Y_k_* denotes the dissipation of *k* due to turbulence (m^2^/s^3^); *Y_ω_* denotes the dissipation of *ω* due to turbulence (s^−2^); *S_k_* is a user-defined source item (m^2^/s^3^); *S_ω_* is a user-defined source item (s^−2^); *G_b_*, *G_ωb_* denotes the buoyancy term (m^2^/s^3^, s^−2^).

Species transport equation:

(8) $$\frac{𝜕}{𝜕t}\left(\rho Y_{i}\right)+\nabla \left(\rho \nu Y_{i}\right)=-\nabla J_{i}$$

where $Y_{i}$ represents the mass fraction of the i-th species (−);ν denotes the velocity vector (m/s); $J_{i}$ indicates the turbulent diffusion rate of the i-th species (m^2^/s).

Real gas state equation (Peng-Robinson):

The Peng-Robinson equation of state is a powerful tool for describing the behavior of real gases, especially under high-pressure conditions (*p* > 2 MPa) [41,42]. It provides more accurate predictions than the ideal gas law by considering molecular volume and intermolecular attractions.

(9) $$p=\frac{RT}{\upsilon -b}-\frac{a\alpha \left(T\right)}{\upsilon \left(\upsilon +b\right)+b\left(\upsilon -b\right)}$$

where v denotes the molar volume (m^3^·mol^−1^), a and b indicate the substance-specific van der Waals parameters (Pa·m^6^·mol^−2^ and m^3^·mol^−1^, respectively), and *α(T)* is the temperature-dependent dimensionless correction factor (−).

### 2.2. Geometric Model and Boundary Conditions

**Figure 1:**
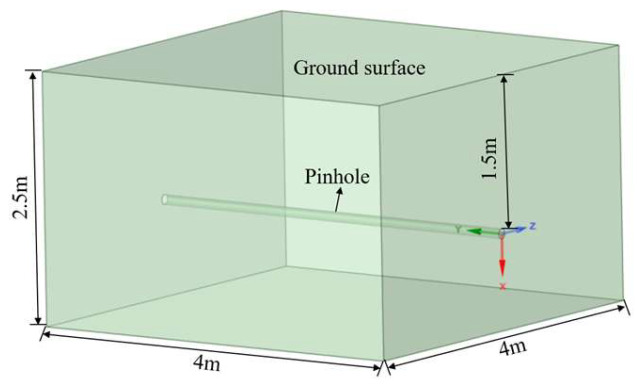
Schematic of the computational domain for a buried hydrogen pipeline.

The computational domain (4 m × 4 m × 2.5 m) was constructed using ANSYS SpaceClaim 2023R2, incorporating a 100 mm diameter pipeline consistent with China’s existing hydrogen transmission infrastructure (Figure 1). The boundary conditions were configured as follows: (1) Pressure-inlet: Orifice surface (stagnation pressure boundary); (2) Pressure-outlet: Domain external boundaries (atmospheric pressure); (3) No-slip wall: Pipeline wall; (4) Porous medium interface: Soil region (isotropic homogeneity assumed). The multiphase transport model accounted for hydrogen-air mixtures within initially air-saturated pores. The porous media formulation incorporated three key parameters: viscous resistance coefficient (α), inertial resistance coefficient (C_2_), and porosity (*φ*). Twenty-eight distinct operational scenarios were systematically designed (Table 3 and Table 4) to investigate parametric dependencies.

### 2.3. Numerical Solution Methodology

**Figure 2:**
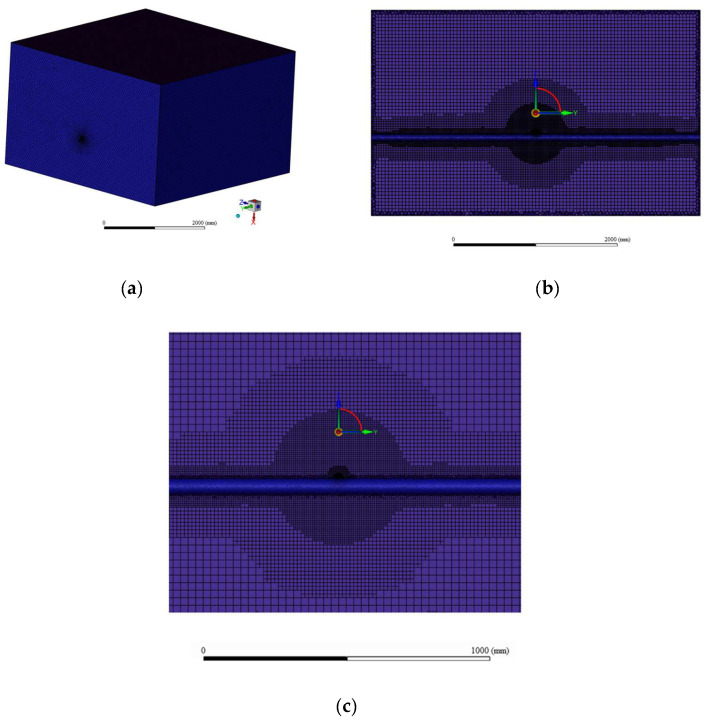
Computational mesh. (**a**) Global mesh topology. (**b**) Cross-sectional mesh refinement. (**c**) Partial enlarged view.

The numerical simulations were performed using the finite-volume-based solver ANSYS Fluent 2023R2. An unstructured polyhedral mesh scheme was implemented with intense local refinement near the orifice (minimum cell size = 0.2 mm, representing 1/20th of a typical 4 mm orifice to resolve the jet dynamics), exhibiting progressive coarsening outward from the leakage source (Figure 2).

An unstructured polyhedral mesh scheme was implemented with local refinement near the orifice (minimum cell size = 0.2 mm), exhibiting progressive coarsening outward from the leakage source (Figure 2). The fine mesh was necessary to resolve the critical physics of the leakage jet (1/20th of the 4 mm orifice) and the macroscopic flow through the porous medium, rather than individual soil pores. Mesh sensitivity was evaluated through four systematically refined grids (approximately 0.4, 1.0, 1.3, and 2.1 million cells).

The convergence was initially assessed by monitoring the hydrogen molar fraction at a key point downstream of the leak and the total mass flow rate at the orifice, both of which are integral parameters for risk assessment. To provide a more robust validation of the solution field’s independence from grid resolution and to address the potential limitation of a single-point metric, the study was expanded. The spatial distribution of the hydrogen dispersion was evaluated by calculating the average hydrogen mole fraction along four strategically placed monitoring lines (see Table 5 for coordinates). These lines were designed to capture the development of the hydrogen plume in the soil and at the ground surface.

**Figure 3:**
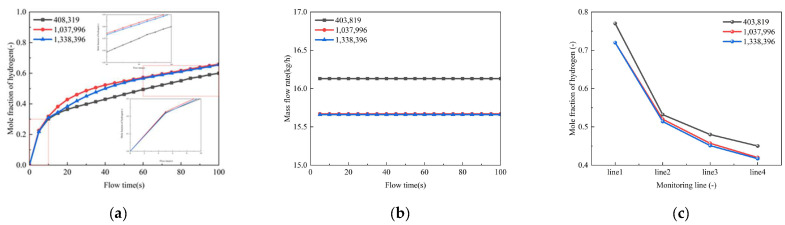
Mesh sensitivity analysis results. (**a**) Temporal evolution of hydrogen mole fraction. (**b**) Mass flow rate through orifice. (**c**) Average hydrogen molar fraction on different monitoring lines.

As shown in Figure 3, both the point monitor and the mass flow rate exhibited convergence with the 1.3-million-cell configuration. Crucially, the line-averaged mole fractions along all four monitoring lines (Figure 3c) also showed negligible variation beyond the 1.3-million-cell mesh. Since the computational cost increases significantly with mesh density while the solution accuracy improves marginally, the mesh system with 1.3 million cells was selected for all subsequent simulations. This comprehensive analysis, utilizing both local and spatially averaged metrics, confirms that the results presented herein are robust and independent of further mesh refinement.

The pressure-based solver was employed with the SIMPLE algorithm for pressure-velocity coupling. The Shear-Stress Transport (SST) *k-ω* model was selected for turbulence closure due to its accuracy for wall-bounded flows. All convective terms were discretized using the second-order upwind scheme. A fixed time step of 0.05 s ensured Courant number stability (Co < 1) [43], with convergence criteria set to 10^−4^ for all residuals.

The time step size was set to a fixed value of 0.05 s. This value was selected based on a preliminary Courant number analysis to ensure numerical stability (Co < 1) [43] throughout the computational domain, particularly in the regions of finest mesh near the leakage orifice. A formal time step sensitivity study was not conducted for this specific work, as the selected time step provided a robust balance between computational efficiency and temporal resolution for the steady-state leakage behavior of primary interest. Future studies focusing on transient initiation phases may benefit from such an analysis.

Convergence was monitored for all governing equations (continuity, momentum, energy, species, etc.). A solution was considered converged when the normalized residuals for all variables fell below the criterion of 10^−4^ and remained stable over subsequent iterations. While stricter criteria (e.g., 10^−6^) are sometimes employed, the chosen criterion of 10^−4^ was deemed sufficient for this study based on two key observations: (1) further reduction in the residuals below 10^−4^ did not yield any perceptible change in the key monitored quantities, namely the mass flow rate at the orifice and the hydrogen concentration at strategic points in the domain; and (2) the solutions exhibited strong numerical stability and conservation properties (e.g., global mass imbalance was consistently below 10^−5^ kg/s), indicating that the solver had reached an acceptable solution. This approach is consistent with established practices for similar large-scale, steady-state CFD simulations, where tracking residual levels to machine zero is computationally expensive and unnecessary for engineering accuracy.

**Figure 4:**
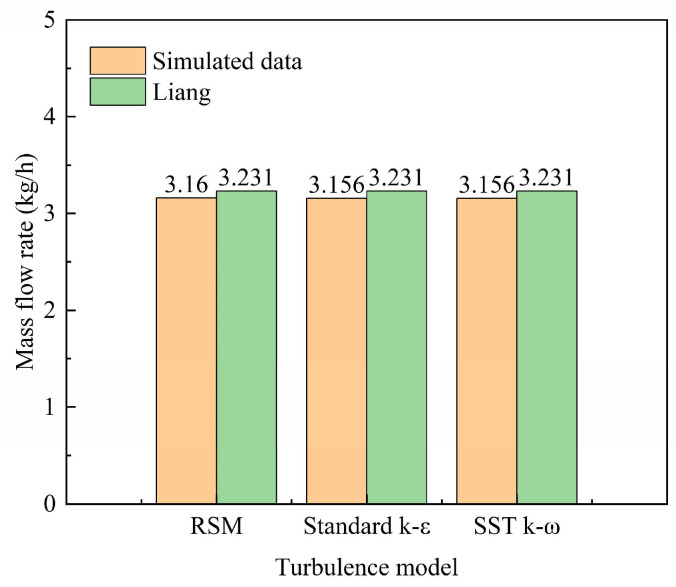
The comparison of an appropriate turbulence model. The data marked in green in the figure is from Liang [37].

The selection of an appropriate turbulence model is critical for accurately capturing the complex flow dynamics of hydrogen jet dispersion and transport through porous soil. To ensure simulation accuracy and justify the model choice, a comparative analysis was conducted among three prevalent models (the Reynolds Stress Model (RSM), the standard *k–ε* model, and the Shear Stress Transport (SST) *k–ω* model) and the experiment [37], as shown in Figure 4. All three models yielded consistent predictions for the integral macroscopic parameter, the hydrogen mass flow rate, with results of 3.16 kg/h, 3.156 kg/h, and 3.156 kg/h for the RSM, standard *k–ε*, and SST *k–ω* models, respectively. This convergence confirms the reliability of the macroscopic predictions for risk assessment purposes. However, significant differences emerged in the resolution of flow details and computational performance: The Reynolds Stress Model (RSM), which solves transport equations for each Reynolds stress component, provides superior capability in capturing anisotropic turbulence effects. Nevertheless, it proved to be computationally expensive and prone to instability within the complex porous media environment of this study. The standard *k-ε* model demonstrated robust computational efficiency. However, its inherent isotropic viscosity assumption led to an over-prediction of turbulent diffusion, which is a significant drawback for modeling buoyancy-driven jets like hydrogen. The SST *k–ω* model effectively blends the robustness of the k-ε model in the far-field with the accuracy of the *k–ω* model in near-wall regions. It delivered superior precision for the multi-scale flows present in this problem (encompassing the jet core, shear layer, and flow within soil pores) while maintaining an optimal balance of computational efficiency and numerical stability. Given that the SST *k–ω* model provided the most physically realistic representation of the turbulence characteristics without prohibitive computational cost, it was selected for all subsequent simulations presented in this work.

The mass flow rates of hydrogen leakage in this study are consistently reported in kilograms per hour (kg/h). While the SI base unit for mass flow is kg/s, the use of kg/h is adopted for two primary reasons: First, the calculated leakage rates in this context are typically of a relatively small magnitude (on the order of 10^0^ to 10^2^ kg/h). Expressing these values in kg/s would result in numbers with an excessive number of leading zeros (e.g., 0.000876 kg/s), which is less intuitive for engineering interpretation and comparison, and can impede the clarity of tabulated data. Second, the unit kg/h is a standard and conventional unit for measuring and reporting continuous flow rates in industrial pipeline operations and risk assessment protocols, thereby enhancing the practical relevance and direct applicability of our findings to field engineers and safety planners. All values can be converted to SI units (kg/s) by dividing by 3600.

### 2.4. Numerical Model Validation

The computational model was validated through a two-stage process to ensure its reliability under different scenarios. Experimental validation first followed the buried pipeline leakage experiment of Liang [44], using compressed air in a geometrically scaled setup with the pipeline burial depth *L* = 600 mm and leakage orifice diameter *d*_0_ = 4 mm, the soil particle size *d*_s_ = 0.198 mm, the porosity *φ* = 0.6, the viscous resistance of the porous medium *α* = 2.834 × 10^9^ m^−2^, and the internal resistance C_2_ = 3.273 × 10^4^ m^−1^. The experimental data sourced from Liang [44] (as shown in Table 6) was obtained with a reported measurement uncertainty of approximately ±8.6% (coverage factor k = 2). This uncertainty was primarily associated with the precision of the pressure transducers and mass flow meters employed in their experimental setup. This approach was adopted due to the scarcity of published experimental data for high-pressure hydrogen leakage in buried pipelines and the significant safety challenges associated with conducting such experiments.

**Figure 5:**
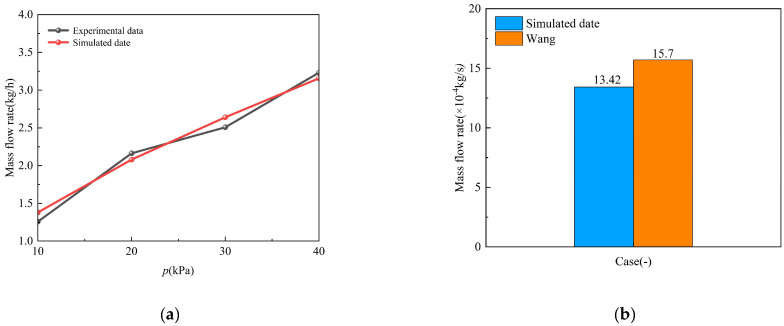
Comparison of Numerical Simulation Results and Experimental Results. (**a**) validation against the experimental data of Liang [44]; (**b**) validation against the experimental data of Wang et al. [45].

Comparative analysis of leakage rates across pressure gradients (10~40 kPa) yielded relative errors of 2.37~8.84% (as shown in Table 6 and Figure 5) within the stated uncertainty bounds (±8.6%) of the experimental measurements themselves. These discrepancies are attributed to soil anisotropy (unaccounted for in the isotropic model), uncontrolled moisture content (assumed dry conditions), and boundary condition idealizations at the experimental setup. The use of compressed air as a surrogate fluid for hydrogen introduces certain limitations that must be acknowledged: (1) Buoyancy Effects: Hydrogen’s low density results in significantly stronger buoyant forces, potentially altering long-term dispersion morphology compared to air. (2) Diffusion Rates: Hydrogen’s higher diffusion coefficient may lead to faster dilution than predicted by the air-validated model. Despite these limitations, this validation successfully benchmarks the model’s core competence in simulating the key physics of pressure-driven jetting and flow through porous media, governed by universal conservation laws. The observed discrepancies are attributed to soil anisotropy, unaccounted moisture, and experimental idealizations.

To directly address the hydrogen-specific behavior and further solidify the model’s validity for hydrogen leakage applications, an additional validation was conducted against the experimental data of Wang et al. [45], which specifically studied hydrogen leakage from long-distance high-pressure buried pipelines. This validation compared the simulated hydrogen mass flow rate against the empirical data under comparable conditions. The results showed a relative error of 14.5% for the hydrogen mass flow rate. This higher error, compared to the air validation, is considered reasonable given the greater experimental complexities associated with high-pressure hydrogen measurements, such as real-gas effects, Joule-Thomson cooling, and the challenges in accurately controlling and measuring small-scale hydrogen flows. The agreement within this margin confirms that the model reliably captures the key aspects of hydrogen dispersion in soil, including the combined effects of buoyancy-driven flow (stronger for H_2_) and porous media resistance. The successful validation against both air and hydrogen experiments comprehensively supports the model’s robustness for the current study. These discrepancies are attributed to soil anisotropy (unaccounted for in the isotropic model), uncontrolled moisture content (assumed dry conditions), and boundary condition idealizations at the experimental setup.

While this validation confirms the model’s accuracy under the specific experimental conditions, its predictive capacity across a broader parameter space—a key concern for comprehensive risk assessment—is thoroughly demonstrated in Section 4.1. As detailed therein, the model was employed to perform an extensive parametric analysis, investigating the influence of critical factors such as pipeline pressure (ranging from 2 to 10 MPa), soil porosity (*φ* = 0.3 ~ 0.6), soil particle size (*d*_s_ = 0.05 ~ 0.2 mm), and leakage orifice diameter (*d*_0_ = 2 ~ 20 mm). The results are discussed in detail in Section 4.1 and Section 4.2. This systematic analysis confirms the model’s robustness and reduces uncertainty regarding its applicability to a wide spectrum of real-world scenarios beyond the direct validation window.

## 3. Parametric Analysis of Hydrogen Dispersion Characteristics

Hydrogen leakage in buried pipelines poses significant safety risks due to its wide explosive range (4–75% *v*/*v*) and low ignition energy [46,47,48]. Current safety protocols establish dual-level detection thresholds: a primary alarm at 25% lower explosive limit (LEL, equivalent to 1% *v*/*v*) and a secondary alarm at 50% LEL (2% *v*/*v*). For risk assessment purposes, subsurface regions exhibiting hydrogen concentrations exceeding 1% *v*/*v* are classified as hazard zones requiring immediate mitigation measures. This section analyzes the influence of eight key factors on hydrogen dispersion, categorized into four groups: leakage hole characteristics, pipeline operational conditions, soil properties, and burial depth. For a complete set of spatial distribution contours that visually illustrate the dispersion patterns under each condition, please refer to the Supplementary Material, Figures S1–S8.

### 3.1. Leakage Dynamics of Buried Hydrogen Pipelines

**Figure 6:**
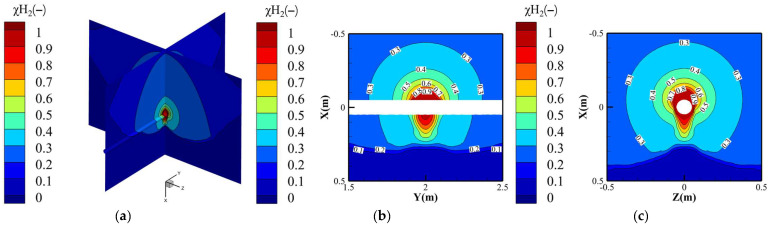
Spatial distribution of hydrogen mole fraction (Condition 3). (**a**) 3D contour; (**b**) Radial profile; (**c**) Axial profile along pipeline.

Figure 6 illustrates the hydrogen molar fraction distribution for Condition 3 (pure hydrogen at 4 MPa operating pressure), exhibiting a characteristic “bulb-shaped” profile (upper spherical zone merging with lower cylindrical region) resulting from coupled leakage dynamics, soil matrix porosity, and gravitational effects. To quantitatively define this morphology, we introduce the aspect ratio of the plume, calculated as the ratio of its axial length to its maximum radial width at a defined concentration threshold. At the Lower Explosive Limit (χH_2_ = 4%), the plume has an aspect ratio of 1.8, confirming its elongation along the vertical axis. This distinct morphology arises from two competing transport mechanisms: (1) Subsonic jet formation: Hydrogen ejection through the 4 mm orifice at 4 MPa operating pressure generates a high-momentum vertical jet, preferentially penetrating soil pores downward to form the cylindrical zone. Soil resistance causes progressive jet deceleration, with axial penetration depth exceeding radial spread. (2) Buoyancy-driven dispersion: Hydrogen’s low density induces rapid upward migration upon jet momentum dissipation. In this regime, molecular diffusion dominates, forming the expanding spherical cloud through Fickian transport.

The velocity field distribution of hydrogen under Condition 3 (4 MPa operating pressure) is presented in Figure 6. Through Peclet number analysis (calculated via Equation (10)), three distinct transport regimes can be identified based on spatial characteristics. The effective diffusion coefficient in soil was determined to be 1.96 × 10^−5^ m^2^/s through modification of the free-space diffusion coefficient *D*_0_ using Equation (11), accounting for soil porosity and tortuosity effects.

(10) $$Pe=\frac{\nu_{c}\cdot L_{c}}{D_{eff}}$$

where *v_c_* represents the characteristic flow velocity (m/s), *L_c_* denotes the characteristic length scale (m), and *D*_eff_ is the effective diffusion coefficient (m^2^/s).

(11) $$D_{eff}=D_{0}\varphi \tau^{-1}$$

where *D*_0_ refers to the free-space diffusion coefficient, with a value of 7.2 × 10^−5^ m^2^/s for hydrogen in air at 300 K. The soil porosity *φ* is 0.3 for Condition 3, while the tortuosity factor *τ* is assumed as 1.1 based on previous studies [49].

**Figure 7:**
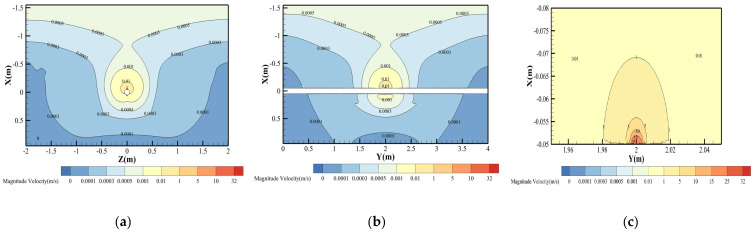
Spatial distribution of velocity (Condition 3). (**a**) Radial profile; (**b**) Axial profile; (**c**) Local enlarged view near the leakage hole.

Near the leakage orifice (*X* ≤ 2.5 mm), hydrogen transport is governed by convective mechanisms. As shown in Figure 7c, the high-pressure hydrogen jet flow adjacent to the orifice reaches a velocity of 32 m/s. The corresponding Péclet number (*Pe* ≈ 6531) significantly exceeds unity (*Pe* » 1), indicating convection-dominated transport that may be accompanied by turbulent dissipation and mechanical energy losses. In deeper soil regions (*X* > 2.5 mm), molecular diffusion becomes the primary transport mechanism. Here, the hydrogen velocity decays sharply to below 0.01 m/s due to pronounced pore resistance, yielding a *Pe* ≈ 2.55 × 10^−3^ (*Pe* « 1), which aligns with Fickian diffusion behavior.

### 3.2. Leakage Hole Characteristic Analysis

#### 3.2.1. Influence of Orifice Diameter

**Figure 8:**
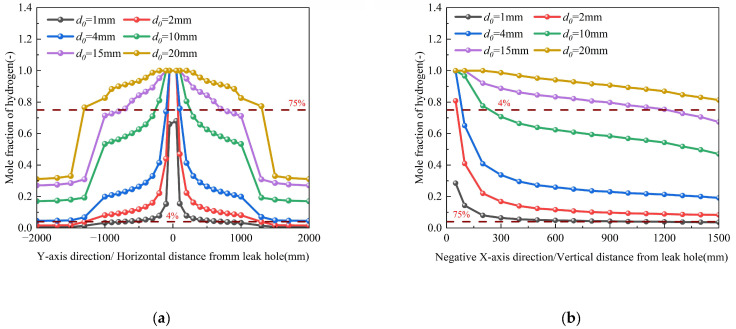
Mole fraction distribution curves under different leakage orifice diameters. (**a**) Along the pipeline length. (**b**) Perpendicular to the leakage orifice.

As detailed in Table 3 (Conditions 1–5), parametric studies were conducted for orifice diameters (*d*_0_) ranging from 1 to 20 mm while maintaining constant boundary conditions. To better capture the nonlinear dispersion behavior, an intermediate 15 mm orifice case was added. The corresponding spatial dispersion contours, which visually demonstrate the expansion of the hazard zone, are provided in Supplementary Figure S1. Numerical simulations reveal two distinct regimes of hydrogen dispersion (Figure 8) based on orifice diameter. In the smaller orifice diameter (*d*_0_ ≤ 2 mm), the increase in the orifice diameter does not significantly change the hydrogen migration range, demonstrating minimal sensitivity to diameter variations. The hydrogen molar fraction gradient along the pipeline axial direction remains below 4.7 × 10^−4^ mm^−1^, while the gradient perpendicular to the pipeline orifice diameter is maintained under 5.39 × 10^−4^ mm^−1^. Conversely, in the larger orifice diameter (*d*_0_ > 2 mm), the hydrogen migration range significantly increases; that is, the hydrogen leakage rate significantly increases. Critically, the relationship between orifice size and hazard extent is strongly nonlinear. As illustrated in Figure 7, the horizontal range where the hydrogen concentration exceeds the Upper explosion limit (UEL) expands dramatically: from approximately ±300 mm for a 10 mm orifice, to ±810 mm for a 15 mm orifice, and further to ±1300 mm for a 20 mm orifice. This more than two-fold increase between 10 mm and 15 mm, compared to the more modest increase from 15 mm to 20 mm, provides valuable insight into a threshold behavior where dispersion dynamics accelerate markedly for leaks beyond a certain critical size. The leakage orifice diameter (*d*_0_) exhibits a quadratic relationship with hydrogen mass flow rate, as the cross-sectional area (A = π*d*_0_^2^/4) increases with the square of the diameter. Consequently, the mass flow rate demonstrates significant enhancement following this geometric scaling principle, expressed mathematically as Equation (1)~(3). Analysis reveals that larger orifice diameters accelerate dispersion velocity through two interrelated mechanisms: (1) substantial reduction in flow resistance (ΔP ~ 1/*d*_0_^4^), (2) strengthened buoyancy effects (The dimensionless group Gr/Re^2^ (ratio of buoyancy to inertial forces) significantly exceeds unity, indicating dominant buoyancy-driven transport in the hydrogen dispersion process). The nonlinear growth of the hazardous zone underscores the disproportionate increase in risk associated with larger pipeline breaches, a key finding for safety perimeter design.

#### 3.2.2. Influence of Orifice Orientation

**Figure 9:**
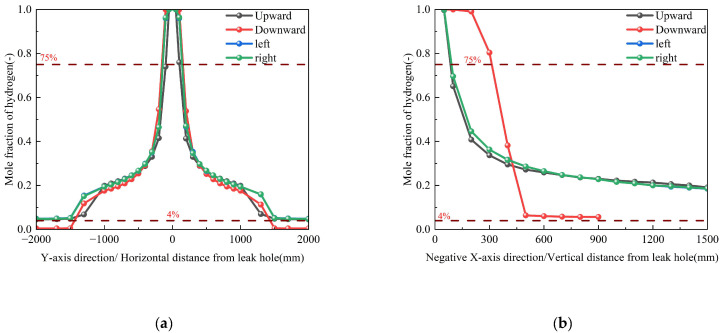
Mole fraction distribution curves of hydrogen under different leakage orifice orientations (upward, downward, leftward, rightward). (**a**) Along the pipeline length. (**b**) Perpendicular to the leakage orifice.

Numerical simulations were conducted to systematically evaluate the influence of orifice orientation (upward, downward, leftward, and rightward) on hydrogen dispersion patterns while maintaining identical boundary conditions (Table 3, Conditions 3 and 6–8). The results demonstrate two distinct characteristic features of the dispersion behavior. First, analysis of horizontal dispersion (as shown in Figure 9a) reveals orientation-independent characteristics, with minimal variation (<5%) in concentration profiles. And the peak concentrations remain consistent across simulation conditions, with spatial variations in their occurrence positions confined within a 50 mm range. Second, buoyancy effects dominate vertical transport, with the upward-facing orifice exhibiting greater vertical dispersion compared to the downward orientation, attributable to hydrogen’s low density (as shown in Figure 9b). The corresponding spatial dispersion contours, which visually demonstrate the expansion of the hazard zone, are provided in Supplementary Figure S2.

### 3.3. Pipeline Transportation Characteristics Analysis

#### 3.3.1. Influence of Pipeline Pressure

**Figure 10:**
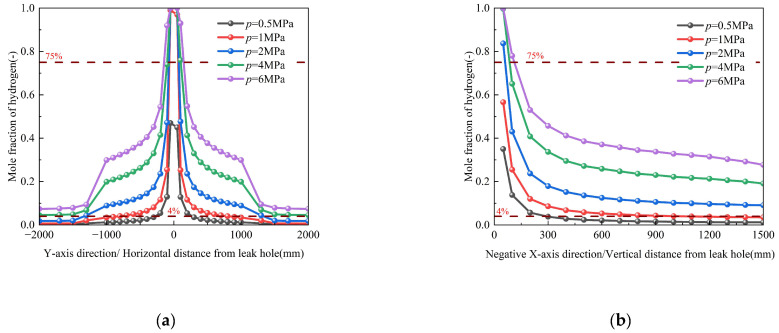
Mole fraction distribution curves of hydrogen under different pipeline pressures. (**a**) Along the pipeline length. (**b**) Perpendicular to the leakage orifice.

**Figure 11:**
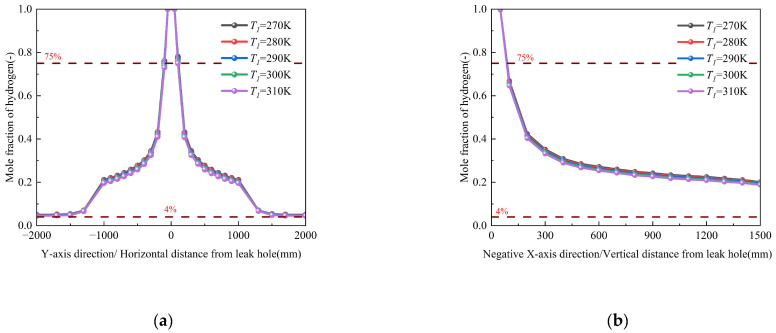
Mole fraction distribution curves of hydrogen under different temperatures. (**a**) Along the pipeline length. (**b**) Perpendicular to the leakage orifice.

Current operational data from Chinese hydrogen pipelines indicate standard delivery pressures ranging from 2.5 to 4 MPa. To comprehensively investigate pressure effects on leakage dynamics, we conducted numerical simulations across five pressure conditions (0.5, 1, 2, 4, and 6 MPa) while maintaining constant environmental parameters (Conditions 3, 12–15). The results reveal three distinct pressure-dependent phenomena, as illustrated in Figure 10. First, elevated pressures (≥2 MPa) substantially enhance hydrogen dispersion, as evidenced by two key observations: (1) the horizontal distance required to reach the secondary alarm threshold (2% *v*/*v*) expands by more than 3.5 times compared to low-pressure conditions (≤1 MPa), and (2) all monitoring positions above the orifice exceed the secondary alarm concentration. Secondly, the leakage mass flow rate exhibits a proportional relationship with pipeline pressure. Figure 11 quantitatively demonstrates the pressure-dependent expansion of explosion hazard zones (defined as 4~75% *v*/*v* hydrogen concentration). At a baseline pressure of 1 MPa, the hazardous area extends horizontally from ±100 to ±300 mm and vertically from 45 to 300 mm, covering an approximate area of 10.2 dm^2^. When the pressure doubles to 2 MPa, the hazard zone expands dramatically to ±100~±1000 mm horizontally and 45~800 mm vertically, resulting in a thirteen times increase in total hazardous area (135.9 m^2^). Furthermore, the hazardous area exhibits nonlinear expansion with increasing pressure, demonstrating accelerated growth at 4 MPa followed by diminished marginal expansion at 6 MPa. These results demonstrate a nonlinear, pressure-dependent expansion of the hazardous zone area, which follows a characteristic saturation growth pattern.

The nonlinear expansion of the hazardous area with increasing pressure is governed by a multiscale coupling of three dominant physical mechanisms that sequentially emerge across different pressure ranges: turbulence-enhanced dispersion dominates at moderate pressures (2–4 MPa) where intensified turbulent mixing significantly enhances hydrogen entrainment and radial spreading, followed by a buoyancy–drag equilibrium regime (4–6 MPa) where the competing effects of buoyant uplift and flow resistance establish dynamic balance leading to stabilized dispersion patterns, and ultimately transitioning to pore-scale flow limitation (>6 MPa) where the dominant flow resistance through soil macropores restricts further expansion and causes saturation of the hazardous area growth. These findings necessitate the implementation of pressure-adaptive safety measures. For systems operating above 2 MPa, additional protective measures such as increased monitoring frequency and expanded exclusion zones should be implemented to account for the nonlinear growth of potential hazard regions. The empirical data further suggest the importance of pressure management in hydrogen pipeline safety design. The corresponding spatial dispersion contours, which visually demonstrate the expansion of the hazard zone, are provided in Supplementary Figure S3.

#### 3.3.2. Influence of Pipeline Temperature

Numerical simulations investigating pipeline temperatures from 270 K to 310 K (Conditions 3 and 19–22, Table 3) reveal complex thermo-fluidic interactions governing hydrogen dispersion, as shown in Figure 11. The results indicate that while temperature exerts a statistically non-significant effect on overall hydrogen dispersion, the magnified view reveals an inverse correlation between temperature and diffusion characteristics—specifically, elevated temperatures reduce the diffusion rate and decrease the total leakage mass. This inverse relationship stems from competing physical mechanisms: although increased temperature enhances molecular kinetic energy, the concomitant density reduction intensifies buoyant uplift while suppressing turbulent mixing through a decreased Reynolds analogy parameter. These counteracting effects collectively modify the concentration field, yet the spatial distribution of hydrogen mole fractions remains statistically invariant across the 270~310 K temperature range. The corresponding spatial dispersion contours, which visually demonstrate the expansion of the hazard zone, are provided in Supplementary Figure S4.

### 3.4. Soil Property Characteristics Analysis

#### 3.4.1. Influence of Soil Porosity

**Figure 12:**
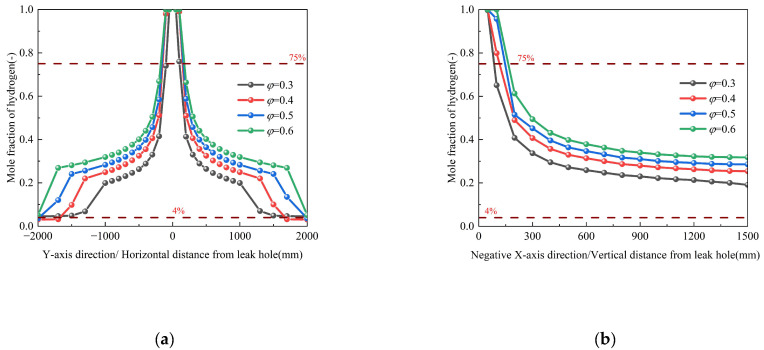
Mole fraction distribution curves of hydrogen under different porosities. (**a**) Along the pipeline length. (**b**) Perpendicular to the leakage orifice.

Soil porosity (*φ*) critically governs hydrogen transport through porous media by directly modulating both the inertial (C_2_) and viscous (*α*) resistance coefficients (Conditions 3, 26–28). Figure 12 reveals two porosity-dependent phenomena: (1) axial concentration profiles maintain quasi-normal distributions despite increasing *φ*, and (2) enhanced dispersion kinetics—where *φ* increases from 0.3 to 0.6—elevate the radial diffusion rate and expand the hazard zone area. These effects arise from the inverse relationship between *φ* and flow resistance coefficients, which reduce momentum dissipation in high-porosity soils. The corresponding spatial dispersion contours, which visually demonstrate the expansion of the hazard zone, are provided in Supplementary Figure S5.

#### 3.4.2. Influence of Soil Particle Size

**Figure 13:**
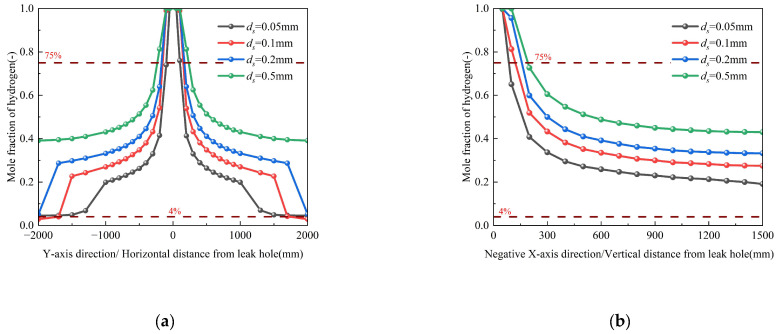
Variation curve of hydrogen mole fraction under different soil particle sizes. (**a**) Along the pipeline length. (**b**) Perpendicular to the leakage orifice.

Numerical simulations were conducted to systematically evaluate the effect of soil particle size (*d*_s_= 0.05~0.5 mm) on hydrogen dispersion characteristics while maintaining constant porosity (*φ* = 0.3) and other boundary conditions (Conditions 3, 23–25 in Table 3). Figure 13 demonstrates that increasing *d*_s_ from 0.05 mm to 0.5 mm enhances hydrogen accumulation, expanding the hydrogen dispersion distances both axially along the pipeline and longitudinally perpendicular to the leakage orifice. This phenomenon arises from the dual control mechanism of pipeline residual momentum and buoyancy–drag equilibrium on hydrogen dispersion. Specifically, increased particle diameter (*d*_s_) reduces the effective pore radius (*r*_eff_) of the soil matrix, thereby decreasing the capillary threshold pressure (*P*_c_ 1/*d*_s_ according to the Young-Laplace equation). This reduction in *P*_c_ simultaneously diminishes both inertial resistance and viscous resistance, ultimately attenuating momentum dissipation. The corresponding spatial dispersion contours, which visually demonstrate the expansion of the hazard zone, are provided in Supplementary Figure S6.

#### 3.4.3. Influence of Soil Temperature

**Figure 14:**
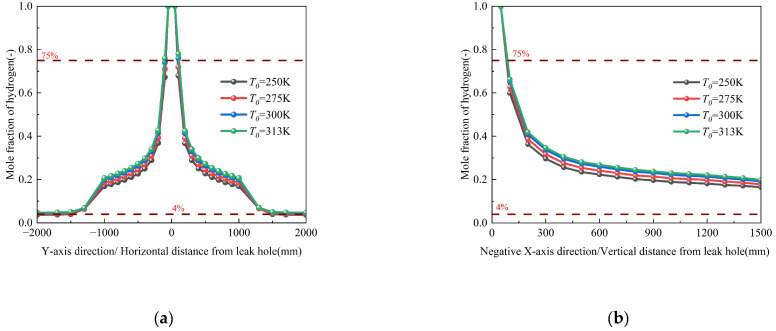
Variation curve of hydrogen mole fraction under different soil temperatures. (**a**) Along the pipeline length. (**b**) Perpendicular to the leakage orifice.

Numerical simulations evaluating seasonal temperature variations (250–313 K, Conditions 3 and 16–18) reveal that soil temperature exerts a limited influence on hydrogen dispersion, with a ≤25% increase in hydrogen mole fraction observed across the 63 K temperature range (Figure 14). This is because the hydrogen leakage process occurs over a significantly shorter duration compared to the soil heat transfer process. This attenuated thermal effect stems from: (1) thermally excited molecular motion; and (2) counteracting buoyancy reduction due to density decreases. When the pipeline operating temperature remains constant, the maximum temperature difference between the soil and the pipeline in this study is within 50 K.

However, it must be pointed out that there are limitations to the conclusions of this study. The current results do not take into account other complex dynamic processes closely related to temperature in the field environment, especially the effects of seasonal freeze–thaw cycles and moisture migration. Freeze–thaw cycles can significantly alter the structure, porosity, and permeability of soil, potentially leading to changes in diffusion rates of orders of magnitude. Similarly, the infiltration of water will occupy soil pores, greatly hindering gas diffusion, and it is itself driven by seasonal changes in precipitation and temperature. Therefore, it is inappropriate to directly extrapolate the conclusion of ‘less impact’ to real site conditions. The results of this study should be understood more as revealing the direct physical effects of temperature itself, rather than a comprehensive prediction of complex field environments. The corresponding spatial dispersion contours, which visually demonstrate the expansion of the hazard zone, are provided in Supplementary Figure S7.

### 3.5. Pipeline Burial Depth

**Figure 15:**
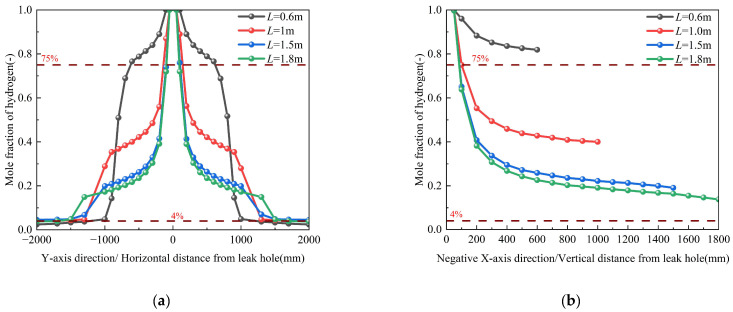
Variation curve of hydrogen mole fraction under different soil burial depths. (**a**) Along the pipeline length. (**b**) Perpendicular to the leakage orifice.

Table 3 presents simulation cases (Conditions 7, 9–11) with pipeline burial depths of 0.6 m, 1 m, 1.5 m, and 1.8 m while maintaining other parameters constant. Figure 15 illustrates the hydrogen mole fraction distribution along both the pipeline length and the direction perpendicular to the leakage orifice at monitoring points for these four burial depths. The results demonstrate two distinct dispersion regimes along the pipeline axis: (1) Within 1000 mm of the leakage point: (i) The high-concentration zone (χH_2_ > 0.4) area expands, (ii) The concentration gradient attenuates progressively and (iii) The diffusion range exhibits moderate expansion (about 33% increase in radial spread), as clearly visualized in the magnified inset of Figure 15a. (2) Beyond 1000 mm: Hydrogen mole fraction distribution becomes depth-independent, indicating transition to Fickian diffusion dominance.

Hydrogen dispersion characteristics perpendicular to the leakage orifice reveal several critical safety implications: (1) Proximal zone (<200 mm from source): Deeper burial depths exhibit greater hydrogen mole fraction attenuation compared to shallow depths. (2) Distal zone (>200 mm): While hydrogen attenuation reaches quasi-steady state, concentrations persistently remain within the hazardous range, necessitating continuous monitoring. (3) Shallow burial hazards: At depths of 0.6 m, sustained hydrogen concentrations (χH_2_ > 0.8) in the soil matrix present acute explosion risks. These conditions facilitate rapid atmospheric migration, with diffusion coefficients 2–3 times higher than deeper burial scenarios. (4) Universal alarm thresholds: All configurations exceeded the alarm threshold within 100 s, highlighting the need for improved early detection systems. This phenomenon primarily stems from two depth-dependent mechanisms: (1) elevated soil resistance, and (2) hindered atmospheric escape pathways (requiring hydrogen to traverse 3–5 times longer capillary pathways). These synergistic effects collectively reduce the effective diffusion coefficient. The retardation follows a power-law relationship with burial depth. These factors work together to slow down the diffusion rate of hydrogen in deeper soil. The corresponding spatial dispersion contours, which visually demonstrate the expansion of the hazard zone, are provided in Supplementary Figure S8.

### 3.6. Implications for Engineering Practice and Safety Distances

The simulated extent of the hydrogen lower explosive limit (based on 4% LEL) for all parameter variations is systematically summarized in Table 7. The results provide direct insights for pipeline risk management and safety engineering. The maximum hazard distances are governed by a complex interplay of factors, with orifice diameter and pipeline pressure identified as the most dominant parameters. For instance, an increase in orifice diameter from 1 mm to 4 mm at 4 MPa pressure expands the horizontal hazard distance from 0.732 m to the domain boundary of 2.000 m.

A key finding for emergency response and monitoring is the critical influence of leak orientation. A downward-oriented leak results in a more confined hazard zone (1.439 m horizontal, 0.900 m longitudinal) due to the impediment of soil, while an upward leak allows buoyant hydrogen to migrate rapidly to the surface, creating a larger aerial extent that reached the model’s geometrical limits (2.000 m horizontal, 1.500 m longitudinal). This underscores the necessity of deploying gas sensors not only along the pipeline route but also at the above-ground level along the right-of-way.

The saturation of values at 2.000 m (horizontal) and 1.500 m (longitudinal) indicates that for high-consequence scenarios (e.g., *d*_0_ ≥ 4 mm, *p* ≥ 2 MPa), the explosive limit reached the physical boundary of our computational domain. This is a crucial result for regulators and engineers, as it signifies that hazard zones for high-pressure hydrogen leaks can be extensive and can easily traverse the typical width of a pipeline easement. Therefore, our data supports the recommendation for conservative safety distances and the implementation of continuous monitoring strategies in areas where high-pressure hydrogen pipelines are present.

The analysis reveals two characteristic dispersion patterns for hydrogen escaping from buried pipelines: (1) a symmetrical distribution along the pipeline axis, and (2) an anisotropic decay profile perpendicular to the leakage orifice, exhibiting rapid initial concentration decline transitioning to gradual attenuation. The dispersion dynamics are governed by five key parameters exhibiting distinct mechanistic relationships. For leakage characteristics, the hydrogen mole fraction (χH_2_) exhibits: (i) a quadratic dependence on orifice diameter due to enhanced mass flux; (ii) a nonlinear pressure-dependent dispersion pattern governed sequentially by turbulence enhancement (2–4 MPa), buoyancy–drag equilibrium (4–6 MPa), and pore-scale flow limitation (>6 MPa). (iii) Soil properties exert equally significant control over hydrogen dispersion; increased porosity elevates χH_2_ through reduced flow resistance. (iv) particle diameter inversely modulates capillary effects. (v) Most notably, deeper burial depth achieves greater reduction in surface-accessible leakage compared to shallow configurations. These parametric relationships collectively determine the hydrogen dispersion profile through coupled fluid-porous media interactions. The extensive set of simulation results, including all distribution contours, is available in the Supplementary Material for further reference.

## 4. Quantitative Analysis on Hydrogen Leakage and Diffusion Dynamics

Building upon the preceding findings, this study identifies pipeline operating pressure, soil porosity, soil particle diameter, and leakage orifice diameter as dominant factors influencing buried hydrogen pipeline leakage, while environmental temperature, leakage temperature, burial depth, and orifice orientation exhibit secondary effects on leakage mass flow rate. We focus our quantitative investigation on these primary determinants.

### 4.1. Parametric Control of Leakage Rate

**Figure 16:**
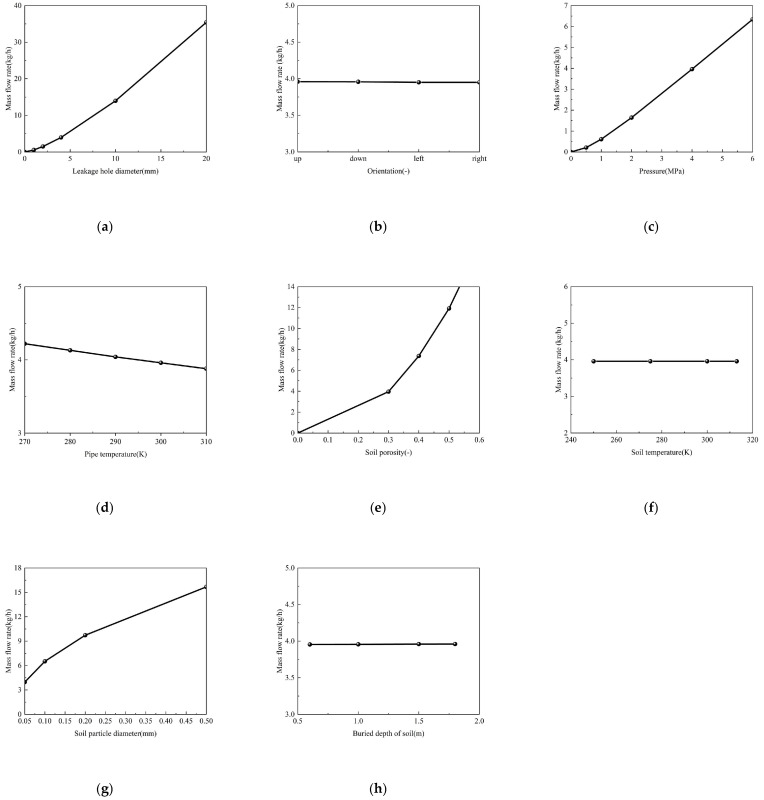
Mass flow rate characteristics under varying leakage and soil conditions. (**a**) leakage orifice diameters; (**b**) leakage orifice orientations; (**c**) pipeline pressures; (**d**) pipeline temperatures; (**e**) soil porosities; (**f**) soil temperatures; (**g**) soil particle sizes; (**h**) burial depths.

In probabilistic risk assessment (PRA) of buried hydrogen pipelines, the mass flow rate (*Q*) at the leakage orifice serves as the pivotal source term parameter that fundamentally governs worst-case release magnitude, atmospheric entrainment efficiency, and flammable cloud formation dynamics [50,51]. This critical parameter intrinsically determines the source strength spectrum, which cascades into dispersion physics, explosion overpressure decay gradients, and safety barrier requirements, making accurate *Q* quantification essential for credible consequence analysis, particularly for high-pressure pipelines (>4 MPa). Our analysis of hydrogen transport through porous media (Section 3) reveals four critical leakage source-term parameters. As demonstrated in Figure 16, the mass flow rate (*Q*) at the leakage orifice shows strong functional dependence on: (1) Orifice diameter (*d*_0_): Directly controls flow area. (2) Pressure (*p*): Primary driving force. (3) Soil porosity (*φ*): Regulates the gas diffusion resistance through permeability. (4) Particle diameter (*d_s_*): Modulates capillary threshold pressure.

As demonstrated in Figure 16, the negligible influence of other factors stems from: (1) Pipeline temperature effects: While Figure 16d shows a measurable decreasing trend in mass flow rate with increasing pipeline temperature—primarily due to reduced gas density at higher temperatures—the magnitude of this effect is secondary compared to the dominant influence of pressure and orifice diameter. This relative insensitivity stems from the fact that rapid gas expansion and Joule-Thomson cooling during the depressurization process at the leak orifice minimize the dependency on the initial temperature condition. Therefore, while non-zero, the temperature effect is a secondary parameter in the context of this large-scale parametric study and does not significantly alter the model’s predictive capability for risk assessment, justifying its exclusion from the final simplified model. (2) Soil temperature effects: Soil temperature exerts a negligible influence on leakage mass flow rate, with its secondary effects through viscosity and saturation. (3) Isotropic dry soil: uniform intrinsic permeability to gas flow attenuates orientation effects on hydrogen dispersion. (4) Pipeline burial depth: Following current industry practice that predominantly adopts natural gas pipeline construction standards (standard burial depth is 1.5 m according to GB 50251-2015 [52]), and building on our Section 3 findings showing minimal leakage variation for depths ≥1.5 m, this study employs a justified simplification by fixing pipeline burial depth at ≥1.5 m for all quantitative leakage analyses. These findings enable model simplification through dimensional analysis, providing a theoretical basis for simplifying the risk assessment model.

**Figure 17:**
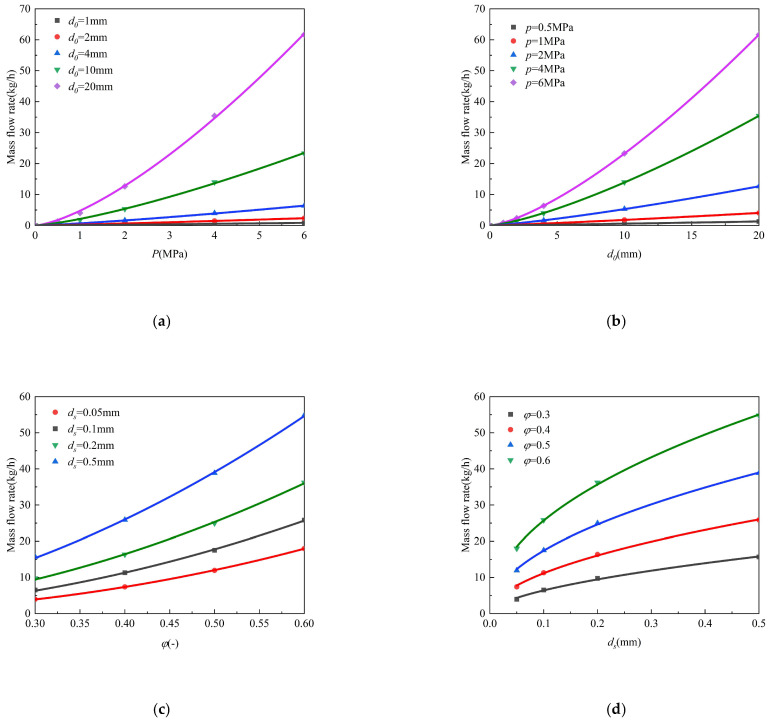
Fitted curve of hydrogen leakage mass flow rate *Q*. (**a**) *p-Q-d_0_* Curve; (**b**) *d_0_-Q-p* Curve; (**c**) *d_s_-Q-φ* Curve; (**d**) *φ-Q-d_s_* Curve.

Figure 17a,b demonstrates that hydrogen leakage rates (*Q*) are primarily governed by pipeline pressure (*p*) and orifice diameter (*d*_0_). Under baseline conditions (*T*_1_ = 300 K, *L* = 1.5 m, *φ* = 0.3, *d_s_* = 0.05 mm), numerical simulations reveal: (i) a superlinear pressure dependence ($Q\propto p^{1.11\sim 1.43}$), with exponents indicating a transition from laminar (1.11 at *d*_0_ = 2 mm) to turbulent-dominated flow (1.43 at *d*_0_ = 8 mm). The correlation coefficients exceed 0.999 for all fitted curves, confirming the robustness of these power-law relationships. and (ii) a superlinear scaling relationship between hydrogen mass flow rate (*Q*) and orifice diameter (*d*_0_), where $Q\propto {d_{0}}^{1.13\sim 1.41}$ with exceptional correlation (*R*^2^ > 0.999). (iii) synergistic enhancement—high pipeline pressure (*p* ≥ 2 MPa) combined with large orifice diameter (*d*_0_ > 10 mm) yields 2.5 times greater *Q* than low-pressure, small orifice diameter conditions.

As shown in Figure 16e,g, soil characteristics significantly modulate leakage behavior. For fixed leakage geometry (*d*_0_ = 4 mm, *p* = 4 MPa), the mass flow rate exhibits (i) strong porosity (*φ*) dependence ($Q\propto \varphi^{1.83\sim 2.20}$) and (ii) moderate particle size sensitivity ($Q\propto d_{s}^{0.47∼0.56}$). Porosity enhancement exerts a more pronounced effect on hydrogen mass flow rate (*Q*) than particle size (*d*_s_)variation. Specifically, at *φ* = 0.6, the leakage rate exceeds that of *φ* = 0.5 conditions even when comparing *d_s_* = 0.1 mm (high-resistance) versus *d_s_* = 0.2 mm (low-resistance) scenarios, indicating porosity-dominated transport (*Q*(*φ* = 0.6, *d_s_* = 0.1 mm) > *Q*(*φ* = 0.5, *d_s_* = 0.2 mm)). All fits show excellent correlation (R^2^ > 0.996), validating the negligible impact of secondary parameters under these experimental conditions.

### 4.2. Developing a Quantitative Leakage-Diffusion Model Using Constrained Optimization

Building upon the factor interaction analysis in Section 4.1, we established a quantitative model to predict the leakage mass flow rate. The model structure is based on the power-law relationship between the mass flow rate (*Q*) and the four dominant parameters identified in Section 4.1:

(12) $$Q=C\cdot d_{0}^{\alpha_{1}}\cdot p^{\beta }\cdot \phi^{\gamma }\cdot d_{s}^{\delta }$$

where C is a scaling constant and *α*_1_, *β*, *γ*, and *δ* are the exponents for orifice diameter (*d*_0_), pressure (*p*), porosity (*ϕ*), and particle diameter (*d_s_*), respectively.

The initial guesses and bounds for the exponents in the optimization were rigorously derived from our preceding analysis to ensure physical validity and accelerate convergence:

(1) Orifice diameter ($d_{0}^{\alpha_{1}}$): Section 4.1 (Figure 17a,b) revealed a superlinear scaling relationship, with the exponent closely aligning with the theoretical value of 2 for orifice flow, as the leakage area is proportional to $d_{0}^{2}$. Consequently, the initial guess for *α*_1_ was set to 2.0 with bounds of [1.8, 2.2].
(2) Pressure ($p^{\beta }$): The analysis in Section 4.1 (Figure 17a,b) indicated a near-linear to slightly superlinear relationship ($Q\propto p^{1.11\sim 1.43}$), transitioning from laminar to turbulent flow regimes. The theoretical expectation for compressible flow through an orifice suggests a value close to 1. Thus, the initial guess for *β* was set to 1.2 with bounds of [1.0, 1.5].
(3) Porosity ($\phi^{\gamma }$): The strong dependence shown in Figure 16e and Figure 17d was used to inform this parameter. The initial analysis suggested a positive correlation stronger than linearity. The initial guess for *γ* was set to 1.5 with bounds of [1.0, 2.0].
(4) Particle size ($d_{s}^{\delta }$): Figure 16g and Figure 17c showed a moderate negative correlation, as increased particle size reduces flow resistance. The initial guess for *δ* was set to −0.5 with bounds of [−1.0, 0].

To precisely determine these exponents, we implement constrained optimization via MATLAB (online)’s fmincon solver, which minimizes the residual sum of squares (RSS) between modeled and experimental mass flow rates as in Equation (13). The initial guess for the optimizer was set to the midpoint of the predetermined bounds for each exponent. This strategy was chosen to avoid initial bias and to facilitate stable convergence.

(13) $$RSS={\sum_{i=0}^{N}\left|Q_{i}-\left(C\cdot d_{0,i}^{\alpha_{1}}\cdot p_{i}^{\beta }\cdot \phi_{i}^{\gamma }\cdot d_{s,i}^{\delta }\right)\right|}^{2}$$

**Figure 18:**
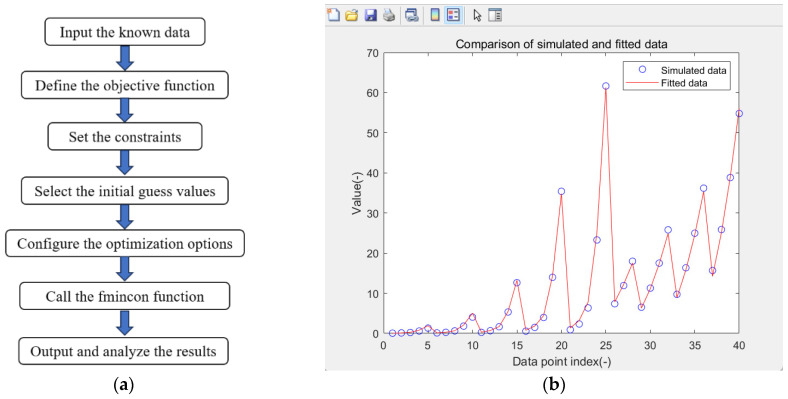
Computational results of the hydrogen leakage model. (**a**) Computational workflow. (**b**) Validation of the optimized power-law model.

The Sequential Quadratic Programming (SQP) algorithm was employed to solve this constrained nonlinear least-squares problem, leveraging its ability to handle high-dimensional parameter spaces through iterative quadratic approximations of the objective function and linearized constraints. Convergence was ensured by setting strict tolerance thresholds (function tolerance < 1 × 10^−6^, parameter tolerance < 1 × 10^−5^), with parallel computing acceleration. The optimized exponents and corresponding RSS values were subsequently analyzed (Figure 18), demonstrating excellent agreement with numerical data (*R*^2^ > 0.98). Furthermore, the optimization process was tested from multiple initial points within the bounds to verify that the solution consistently converged to the same global minimum, confirming the robustness of the identified parameters.

**Figure 19:**
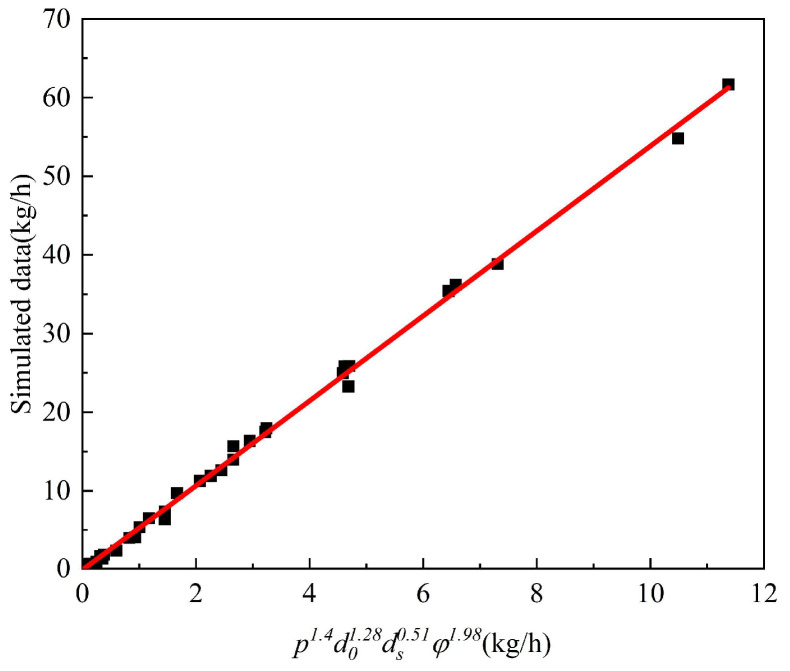
Fitted Curve Plot.

The optimal exponents derived through MATLAB optimization were determined as *α*_1_ = 1.28 (leakage orifice diameter, *d*_0_), *β* = 1.4 (pipeline pressure, *p*), *γ* = 1.98 (soil porosity, *φ*), and *δ* = 0.51 (soil particle diameter, *d_s_*). Figure 19 demonstrates excellent agreement between the fitted curve and all simulated conditions from Section 4.1, with a correlation coefficient of R^2^ = 0.998. The resulting power-law equation for hydrogen leakage mass flow rate (*Q*) is given by:

(14) $$Q=5.372p^{1.4}d_{0}^{1.28}d_{s}^{0.51}\varphi^{1.98}$$

### 4.3. Physical Interpretation of the Model Exponents

While the optimization-derived exponents provide an excellent statistical fit, their values are not arbitrary but reflect the underlying physics of compressible flow through porous media. Here, we interpret each exponent through the lens of fluid dynamics:

#### 4.3.1. Exponent for Orifice Diameter α_1_

The value of 1.28 deviates from the theoretical exponent of 2.0 for ideal orifice flow (where *Q* ∝ *A* ∝ *d*_0_^2^). This discrepancy is physically meaningful and arises from scale-dependent flow resistance effects. For small diameters (*d*_0_ ≤ 2 mm as identified in Section 3.2.1), viscous friction and the vena contracta effect (flow contraction past the orifice) become increasingly significant, reducing the effective flow area and causing the flow rate to scale with *d*_0_^1.28^ rather than *d*_0_^2^. This exponent effectively captures the transition towards laminar-dominated flow resistance at smaller leakage scales.

#### 4.3.2. Exponent for Pressure β

The value of 1.4 exceeds the theoretical value of 1.0 for isenthalpic (Joule-Thomson) choked flow of an ideal gas. This indicates that the real-gas expansion process within the soil environment is not perfectly isenthalpic. The higher exponent suggests additional work is done against the porous matrix, and that frictional heating and local thermal effects slightly counteract the Joule-Thomson cooling, maintaining a higher driving force and resulting in a stronger-than-linear dependence on upstream pressure.

#### 4.3.3. Exponent for Pressure γ

The exponent of 1.98, very close to 2, is highly significant. It indicates that the leakage rate is nearly proportional to the square of the porosity (*Q* ∝ *φ*^2^). This strong dependence suggests that porosity is the primary factor controlling the effective permeability of the soil matrix to gas flow. The value aligns with the notion that increased porosity not only expands the flow area but also dramatically reduces flow path tortuosity, thereby facilitating a much higher mass flow rate.

#### 4.3.4. Exponent for Particle Diameter d_s_

The exponent of approximately 0.51 suggests a relationship where the flow rate scales roughly with the square root of the particle diameter ($Q\propto \sqrt{d_{s}}$). This reflects the complex role that particle size plays in controlling permeability, which often follows a Kozeny-Carman type relationship. The fitted exponent of 0.51 indicates that while larger particles increase permeability (and thus flow rate), the relationship is moderated by other factors, such as the compaction and arrangement of the soil grains, which can influence tortuosity and pore connectivity independently of the primary particle size.

This physical interpretation transforms the empirical correlation into a semi-empirical model whose parameters are not merely statistical but are grounded in the physics of compressible flow and porous media transport.

### 4.4. Model Validation

#### 4.4.1. Results of Model Validation

The predictive accuracy of the hydrogen leakage-diffusion model was validated through a multi-faceted approach encompassing extended parametric comparison and performance evaluation against existing models.

Firstly, the model was validated against an expanded set of 17 distinct operating conditions (see extended Table 8), which notably incorporated critical variations in orifice diameter (3, 7, 15, and 16 mm). A comparative analysis between simulated and theoretically calculated mass flow rates—derived from Equation (14)—is presented in Table 8, revealing absolute errors ranging from 0.88% to 24.36%, with an overall mean absolute error (MAE) of 10.2%. This MAE, along with the excellent agreement (errors consistently below 3.5%) observed for the newly introduced larger orifice diameters (15 mm and 16 mm) under moderate-to-high pressure conditions, demonstrates the model’s capability to accurately capture parametric dependencies of hydrogen leakage rates across a broad range of realistic release scenarios. The higher errors are primarily associated with low-pressure conditions (e.g., 0.5 MPa), which pose challenges both numerically and theoretically (as reflected in Equation (14)), yet correspond to a less critical regime in the context of high-pressure pipeline safety assessment.

**Figure 20:**
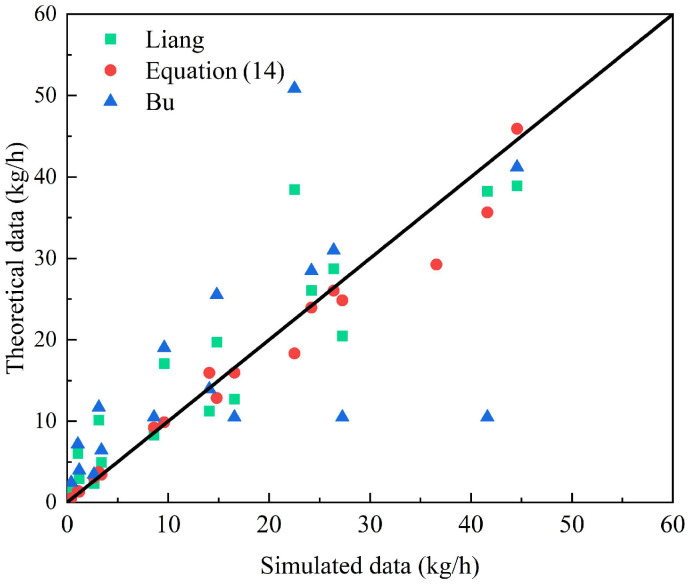
Comparison of fitted and simulated results with error quantification. The data marked in green rectangle in the figure is from Liang [37]. The data marked in blue triangle in the figure is from Bu [35].

To further evaluate the predictive accuracy of the proposed model (Equation (14)), a comparative analysis was performed against two widely referenced models in the field—those developed by Liang et al. [37] and Bu et al. [35], as showed in Table 9 and Figure 20. As summarized in Table 9, the proposed model exhibits significantly improved agreement with theoretical expectations across a broad spectrum of conditions, particularly under elevated pressure regimes (2–6 MPa) and diverse soil properties.

Notably, the mean absolute error (MAE) of Equation (14) is 12.15%, indicating very good overall accuracy. This performance must be interpreted within the context of engineering applications, especially those involving safety-critical systems. It is essential to highlight that the models by Liang and Bu show systematic and substantial over-predictions, often exceeding 100% under conditions of high porosity or larger orifice sizes. For example, at a porosity of 0.6, the proposed model predicts a leakage rate of 29.24 kg/h, whereas the models of Liang and Bu predict 66.48 kg/h and 89.33 kg/h, respectively. Such deviations could lead to overly conservative and economically inefficient safety designs.

In pipeline hazard assessment, the acceptable tolerance for predictive models is not governed by a universal threshold but rather depends on the specific application context and the degree of conservatism incorporated into subsequent risk mitigation strategies. An MAE of approximately 12% is generally deemed acceptable for complex multiphase flow problems in porous media—particularly when the model shows no systematic bias and captures dominant physical trends accurately. Furthermore, the superior performance of the proposed model over existing alternatives underscores its practical utility for risk quantification and scenario screening in the design and monitoring of buried hydrogen pipelines.

#### 4.4.2. Discussion on Model Accuracy and Engineering Applicability

Validation against numerical data reveals two distinct parameter regimes governing the predictive accuracy of the proposed empirical model, as summarized in Table 8. This regime-based analysis is critical for defining the model’s operational boundaries and ensuring its appropriate application in risk assessment.

(1) High-Fidelity Regime: Within the recommended operational range (pressure: 1~6 MPa; soil particle diameter: 0.05~0.50 mm; high-porosity: 0.3~0.5), the model demonstrates excellent predictive performance, with a mean absolute error (MAE) <10%. The proposed model demonstrates optimal validity within specific intermediate parameter regimes characterized by: Reynolds numbers (0.1 < Re < 10) indicating weak inertial effects without turbulent flow, Knudsen numbers [53] (Kn < 0.01) ensuring continuum flow conditions, capillary numbers (10^−4^ < Ca < 10^−3^) representing balanced capillary-inertial force interactions, and moderate porosity ranges (0.3 ≤ *φ* ≤ 0.5). Within this regime, the model is both accurate and reliable, making it highly suitable for engineering applications such as quantitative risk assessment (QRA).
(2) High-Error Regime: Under low-pressure (<1 MPa) and high-porosity (*φ* > 0.5) conditions, errors exceed 20%. This is not a failure of the model but a result of its empirical parameters being calibrated within the high-fidelity regime. The high errors signify a fundamental shift in the dominant physics, moving beyond the scope of the current formulation. The primary mechanisms responsible are: (1) Deviation from Continuum Flow at Low Pressure: In low-pressure, small-pore systems (pore diameter < 100 nm), the Knudsen number exceeds 0.01 (Kn > 0.01), marking the entry into the non-continuum slip flow and transitional flow regimes. In this regime, the increasing frequency of molecule-wall collisions (as opposed to intermolecular collisions) leads to flow phenomena that deviate from the standard continuum assumptions underlying the Darcy–Forchheimer model. This phenomenon is compounded by capillary trapping effects, where strong gas–liquid interfacial forces in nanoscale pores can immobilize hydrogen molecules, drastically reducing effective permeability in a way that our macroscopic porosity-permeability relationship cannot capture. (2) Onset of Strong Inertial and Nonlinear Effects at High Porosity: Under high-porosity conditions (*φ* ≥ 0.6), the flow dynamics undergo two key changes. First, the increased permeability leads to higher velocities, making the inertial drag term (modeled by the Forchheimer coefficient) disproportionately large compared to the viscous term, requiring more complex corrections. Second, and more importantly, the soil structure itself changes; porosities above 0.5 often indicate highly interconnected macropore networks. Flow through these channels is not adequately characterized by conventional models that treat the soil as a homogeneous resistive medium. The nonlinear flow behavior in such structures is not fully encapsulated by our chosen power-law formulation.

The clear identification of this high-error regime is itself a valuable outcome. It delineates the domain of model validity and alerts pipeline engineers and risk assessors to exercise caution when applying the model to scenarios involving very low-pressure leaks or highly porous soils. In such cases, more advanced modeling approaches (e.g., pore-scale or multiphase simulations) are recommended. Thus, the proposed model serves as an efficient and accurate tool for the most common risk scenarios within the high-fidelity regime, while also providing a clear framework for identifying situations that necessitate specialized modeling methodologies.

## 5. Conclusions and Prospects

### 5.1. Conclusions

This study systematically investigates pure hydrogen dispersion characteristics in soil environments using ANSYS Fluent simulations, elucidating flow mechanisms at various leakage locations along buried pipelines. Through comprehensive analysis of orifice characteristics, operating conditions, and soil properties, we establish a nonlinear regression model via the Sequential Quadratic Programming (SQP) algorithm for leakage quantification, achieving < 10% MAE in moderate-porosity soils. Key findings reveal:

(1) Hydrogen dispersion exhibits a bulb-shaped profile due to coupled leakage dynamics, soil porosity, and gravitational effects, governed by competing mechanisms of subsonic jetting and buoyancy-driven dispersion.
(2) The study identifies three distinct transport regimes: (i) a near-field zone (*X* ≤ 2.5 mm) characterized by convection-dominated subsonic jets and (ii) a far-field zone (*X* > 2.5 mm) where Fickian diffusion becomes the dominant transport mechanism.
(3) The analysis reveals a critical threshold behavior in orifice diameter effects: while hydrogen dispersion demonstrates minimal sensitivity to diameter variations below 2 mm (*d*_0_ ≤ 2 mm), a marked acceleration in dispersion occurs beyond this threshold (*d*_0_ > 2 mm) through three interrelated mechanisms—(i) substantially reduced flow resistance, (ii) enhanced buoyancy-driven transport, and (iii) significantly shortened time-to-LEL (Lower Explosive Limit) attainment.
(4) Horizontal dispersion remains isotropic, while vertical dispersion demonstrates orientation-dependent buoyancy effects.
(5) The pressure-dependent analysis demonstrates distinct nonlinear characteristics, revealing (i) a proportional relationship between mass flow rate and pressure across the studied range, while (ii) the hazardous area expansion exhibits marked nonlinear behavior when pressure exceeds 2 MPa (*p* ≥ 2 MPa), indicating a threshold effect in risk propagation.
(6) Temperature invariance (270~310K) in molar fraction distribution, though 12% variation in pipe temperature introduces uncertainty.
(7) Porosity (*φ*) increases enhance dispersion without altering axial concentration profiles.
(8) The study demonstrates that increasing soil particle size (*d_s_*) enhances hydrogen accumulation through two coupled physical mechanisms: (i) residual momentum effects from the leaking jet, and (ii) fundamental shifts in the buoyancy–drag equilibrium governing gas transport through porous media.
(9) Based on the established model and assuming that the soil medium remains dry and structurally stable, this study analyzed the effect of temperature on hydrogen diffusion. The results show limited net influence of soil temperature variations on hydrogen dispersion characteristics, as thermally activated molecular motion (enhancing diffusion) is substantially counterbalanced by density-mediated buoyancy reduction (impeding vertical transport).
(10) The study identifies a critical transition in burial depth effects at 1000 mm, with near-field behavior (*L* ≤ 1000 mm) characterized by expanding high-concentration zones (χH_2_ > 0.4) and decaying concentration gradients, while far-field regions (*L* > 1000 mm) exhibit depth-independent dispersion consistent with Fickian diffusion regimes.
(11) A new leakage diffusion model was established through SQP optimization, and the fidelity of continuous flow was very high (MAE < 10%) in medium to high pressure and weak inertia medium diameter soil.

### 5.2. Limitations and Future Work

The findings of this study are subject to the limitations of its underlying model assumptions, which were necessary to establish foundational insights but simplify real-world complexity. The conclusions are therefore most applicable to idealized, well-drained soil conditions. The most significant limitations, as rightly noted, concern the simplification of the soil medium:

(1) Exclusion of Soil Moisture and Humidity: The assumption of completely dry soil is a major simplification, as identified by the reviewer. Groundwater and soil moisture can drastically alter the system’s behavior by: (i) reducing the effective gas-phase porosity and diffusion coefficients, thereby trapping hydrogen and potentially creating localized, high-concentration pockets; (ii) controlling relative permeability, which governs the flow capacity of the gas phase and can lead to preferential flow paths or capillary trapping; and (iii) significantly reducing the lateral spread of the hydrogen plume. The presence of moisture is perhaps the single most critical factor that would differentiate our baseline case from field conditions.
(2) Assumption of Homogeneity and Isotropy: Natural soils are stratified and anisotropic. This simplification neglects the formation of preferential flow paths through high-permeability layers or capillary trapping by low-permeability layers. These heterogeneities can drastically alter dispersion patterns, accelerating hydrogen migration in certain directions while trapping it in others, leading to potential accumulation zones distant from the leak source.
(3) Validation Methodology Using Air: The model was validated against experimental data for compressed air leakage [44], not hydrogen. While this is a common and practical approach due to the scarcity of high-quality, large-scale hydrogen leakage datasets, it introduces limitations. The key differences in physicochemical properties between air and hydrogen—most notably hydrogen’s lower density, higher buoyancy, and higher diffusion coefficient—mean that the validation primarily confirms the model’s accuracy in capturing the flow physics through porous media rather than the exact dispersion patterns of hydrogen.
(4) Non-reactive Transport: While justified for the high-flow, advection-dominated leaks studied here, this assumption may not hold for low-flow, chronic leakage scenarios or in soils with specific biogeochemical properties. Processes like microbial oxidation (which could mitigate hazard) or adsorption (which could create a lingering source term) are not captured.

Subsequent research should therefore prioritize transcending these limitations. A systematic sensitivity analysis incorporating a range of soil moisture contents is identified as the highest priority to strengthen the model’s real-world applicability. Future work must:

(1) Investigate soil moisture effects. A systematic sensitivity analysis incorporating a range of soil water contents is the most critical next step to strengthen the model’s real-world applicability and quantify its impact on dispersion distance, accumulation behavior, and time-to-LEL attainment.
(2) Conduct validation with hydrogen experiments. Where possible, future studies should seek to validate models against controlled hydrogen release experiments to fully confirm predictive accuracy for hydrogen-specific dispersion.
(3) Investigate geological heterogeneity. Future studies should model stratified soil profiles with contrasting permeabilities to investigate capillary trapping and preferential flow, and incorporate soil anisotropy to simulate realistic geological settings.
(4) Incorporate insights from adjacent fields. Learnings from recent underground hydrogen storage (UHS) research [39,40] on long-term hydrogen-geomechanical and geochemical interactions should be integrated into pipeline leakage models.
(5) Ultimately, develop coupled models. Coupling Thermo-Hydro-Mechanical-Chemical (THMC) models will be essential to comprehensively evaluate complex field scenarios, including seasonal effects like freeze–thaw cycles and water infiltration, to provide a more reliable basis for safety assessments under specific climatic and geological conditions.

## Figures and Tables

**Table 1:** Summary of key research studies on leakage and dispersion characteristics in buried hydrogen-related pipelines.

Researcher	Model	Investigated Parameters	Research Methodology
Peng et al. [26]	small hole	Leakage hole size (5, 10, 20 mm), pipeline pressure (0.2, 0.3, 0.4 MPa), pipeline burial depth (0.8, 1.1, 1.4 m), soil type (chalky sand, loam, clay).	Numerical simulation
Hu et al. [27]	small hole	Leakage hole orientations, leakage hole shapes, leakage hole diameters (5, 10, 20 mm), pipeline burial depths (0.8, 1.0, 1.5 m), soil properties (with varying porosities), pipe pressures (0.30, 0.35, 0.40 MPa).	Numerical simulation
Zhang et al. [28]	large hole	Leakage hole size (20, 30, 40 mm), soil type (sand, clay), pipe pressure (1, 2, 4 MPa), pipe diameter (304, 508 mm).	Numerical simulation
Liu et al. [29]	small hole	Soil permeability, wind speed.	Numerical simulation
Taibi Hen et al. [30]	small hole	Pipe pressure (2, 3, 4, MPa), soil type (sandy, loamy, clay), leakage hole diameter (10, 12, 15, 20 mm).	Numerical simulation
Zhu et al. [31] ^1^	small hole	Leakage hole size (1 mm), Pipe pressure (4, 5.8 MPa), pipeline burial depths (1.4 m), leakage hole orientations (12 o’clock and 3 o’clock).	Experimental simulation
Li et al. [1]	small and large holes	Pipe pressure (0.2, 0.3, 0.4 MPa), ground conditions (hardened, unhardened ground), leakage hole size (10, 20, 30 mm), burial depth (1, 1.2, 1.5 m).	Numerical simulation
PHMSA Data [32]	small hole	Pipe diameter: 254 mm, wall thickness: 6.35 mm, material: API 5L X42, leakage pressure: 1.79 MPa, leakage volume: 48.15 × 10^3^ Nm^3^, impact radius: 28.35 m, Leak type: pinhole leak	Accident record

^1^ Zhu et al. [31] investigated natural gas–hydrogen blends, focusing on how the hydrogen blending ratio affects soil temperature and fiber optic detection range. However, their study does not provide specific leakage data—such as mass flow rate, pressure, or velocity—for pure hydrogen (100% H_2_) scenarios. Thus, while informative for blended gases, their results are not directly applicable to pure hydrogen releases.

**Table 2:** Prediction Model for Leakage Rate of Buried Pipelines.

Researcher	Characterization of Predictive Model Characteristics
Ebrahimi-Moghadam et al. [33]	The leakage pressure, leakage orifice diameter, and pipe diameter ratio were considered
Wang et al. [34]	The leakage pressure, leakage orifice diameter, and porosity were considered
Bu [35]	The leakage pressure, leakage orifice diameter, inertial resistance coefficient, and viscous resistance coefficient were considered
Liu et al. [36]	The leakage pressure, leakage orifice diameter, porosity, and soil particle size were considered.
Liang et al. [37]	The leakage pressure, leakage orifice diameter, and pipeline burial depth were considered.

**Table 3:** Parametric matrix for leakage scenarios in buried hydrogen pipelines ^1^.

Condition	Orifice Diameter (*d_o_*)/10^−3^ m	Orifice Orientation (*θ*)/−	Burial Depth (*L*)/m	Soil Type	Pipeline Pressure (*p*)/MPa	Soil Temperature (*T*_0_)/K	Pipeline Temperature (*T*_1_)/K
1	1	Upward	1.5	1	4.0	300	300
2	2	Upward	1.5	1	4.0	300	300
3 †	4	Upward	1.5	1	4.0	300	300
4	10	Upward	1.5	1	4.0	300	300
5	20	Upward	1.5	1	4.0	300	300
6	4	downward	1.5	1	4.0	300	300
7	4	left	1.5	1	4.0	300	300
8	4	right	1.5	1	4.0	300	300
9	4	Upward	0.6	1	4.0	300	300
10	4	Upward	1	1	4.0	300	300
11	4	Upward	1.8	1	4.0	300	300
12	4	Upward	1.5	1	0.5	300	300
13	4	Upward	1.5	1	1.0	300	300
14	4	Upward	1.5	1	2.0	300	300
15	4	Upward	1.5	1	6.0	300	300
16	4	Upward	1.5	1	4.0	250	300
17	4	Upward	1.5	1	4.0	275	300
18	4	Upward	1.5	1	4.0	313	300
19	4	Upward	1.5	1	4.0	300	270
20	4	Upward	1.5	1	4.0	300	280
21	4	Upward	1.5	1	4.0	300	290
22	4	Upward	1.5	1	4.0	300	310
23	4	Upward	1.5	2	4.0	300	300
24	4	Upward	1.5	3	4.0	300	300
25	4	Upward	1.5	4	4.0	300	300
26	4	Upward	1.5	5	4.0	300	300
27	4	Upward	1.5	6	4.0	300	300
28	4	Upward	1.5	7	4.0	300	300

^1^ The highlighted cells indicate the parameter that is varied from the baseline condition (Condition 3, indicated by †).

**Table 4:** Parameter metrics for different soil types of buried pure hydrogen pipelines.

Soil Type	Soil Type (Description)	Porosity (*φ*)/-	Soil Particle Size (*d*_s_) /10^−3^ m	Viscous Resistance Coefficient (*α*)/m^−2^	Inertial Resistance Coefficient (C_2_)/m^−1^
1	Fine Sand	0.3	0.05	1.09 × 10^12^	1.81 × 10^6^
2	Fine Sand	0.3	0.1	2.72 × 10^11^	9.07 × 10^5^
3	Coarse Sand	0.3	0.2	6.8 × 10^10^	4.53 × 10^5^
4	Gravel	0.3	0.5	1.09 × 10^10^	1.81 × 10^5^
5	Packed Fine Sand	0.4	0.05	3.375 × 10^11^	6.56 × 10^5^
6	Loose Fine Sand	0.5	0.05	1.2 × 10^11^	2.8 × 10^5^
7	Very Loose Fine Sand	0.6	0.05	4.4 × 10^10^	1.29 × 10^5^

**Table 5:** Numerical discretization schemes and algorithm selection.

	Line1	Line2	Line3	Line4
Start point/(m)	(−0.05, 2, 0)	(−0.05, 2.3, 0)	(−0.05, 2.6, 0)	(−0.05, 3, 0)
End point/(m)	(−1.55, 2, 0)	(−1.55, 2.3, 0)	(−1.55, 2.6, 0)	(−1.55, 3, 0)

**Table 6:** Conditions and Results for Numerical Models validation.

Leakage Hole Diameter (10^−3^ m)	Leakage Pressure (kPa)	Mass Flow Rate (kg/h)		Absolute Error (%)
		Simulation Results	Experimental Results	
4	10	1.38	1.258	8.84
	20	2.08	2.163	3.99
	30	2.64	2.508	5.00
	40	3.156	3.231	2.37

**Table 7:** Maximum simulated horizontal and longitudinal hazard distances (to LEL) under varying parameters ^1^.

Parameter category (-)	Parameter Value (-)	Maximum Horizontal Distance (m)	Maximum Longitudinal Distance (m)
Orifice Diameter (*d*_0_)/ (×10^−3^ m)	1	0.732	1.027
	2	1.288	1.500
	4, 10, 15, 20	2.000	1.500
Pipeline Pressure (*p*)/MPa	0.5	0.278	0.291
	1	0.833	1.009
	2	1.338	1.500
	4, 6	2.000	1.500
Pipeline Temperature (*T*_1_)/K	270, 280, 290, 310	2.000	1.500
Soil porosity (-)	0.3, 0.4, 0.5, 0.5	2.000	1.500
Soil particle size/ (×10^−3^ m)	0.05, 0.2, 0.5	2.000	1.500
	0.1	1.742	1.500
Soil Temperature (*T*_0_)/K	250	1.500	1.500
	275, 300, 313	2.000	1.500
Burial Depth (*L*)/m	0.6	1.211	1.500
	1	1.843	1.500
	1.5, 1.8	2.000	1.500
Orifice orientation (*θ*)/-	Upward, Left, Right	2.000	1.500
	Downward	1.439	0.900

^1^ The geometrical dimensions of the computational domain were 2.000 m (horizontal) × 1.500 m (longitudinal). Values equaling these limits indicate that the flammable cloud extended to the domain boundary for the given parameter set.

**Table 8:** Comparative validation of hydrogen leakage rates between theoretical predictions and numerical simulations.

Orifice Diameter/ 10^−3^ m	Pressure/MPa	Porosity/-	Soil Particles/ 10^−3^ m	Leakage Rate (kg/h)		Absolute Error (%)
				Equation (14)	Simulated Value	
7	0.5	0.3	0.05	0.49	0.394	24.36
	1	0.3	0.05	1.3	1.19	9.24
	2	0.3	0.05	3.42	3.39	0.88
	4	0.3	0.05	9.23	8.597	7.36
	6	0.3	0.05	15.94	14.086	13.16
	4	0.3	0.1	15.97	16.566	3.56
	4	0.3	0.2	24.84	27.26	8.87
	4	0.3	0.5	35.64	41.63	14.39
	4	0.4	0.05	12.86	14.82	13.22
	4	0.5	0.05	18.32	22.51	18.61
	4	0.6	0.05	29.24	36.576	20.04
16	1	0.3	0.05	3.73	3.128	19.4
	2	0.3	0.05	9.86	9.609	2.64
	4	0.3	0.05	26.03	26.4	1.4
	6	0.3	0.05	45.92	44.569	3.02
3	4	0.3	0.05	3.05	2.657	14.95
15	4	0.3	0.05	23.96	24.194	0.943

**Table 9:** Comparative validation of hydrogen leakage rates between theoretical predictions and other researchers.

Orifice Diameter/ 10^−3^ m	Pressure/MPa	Porosity/-	Soil Particles/ 10^−3^m	Leakage Rate (kg/h)		
				Equation (14)	Liang [37]	Bu [35]
7	0.5	0.3	0.05	0.49	1.75	2.43
	1	0.3	0.05	1.3	2.93	3.95
	2	0.3	0.05	3.42	4.94	6.44
	4	0.3	0.05	9.23	8.31	10.49
	6	0.3	0.05	15.94	11.26	13.95
	4	0.3	0.1	15.97	12.71	10.49
	4	0.3	0.2	24.84	20.45	10.49
	4	0.3	0.5	35.64	38.22	10.49
	4	0.4	0.05	12.86	19.69	25.52
	4	0.5	0.05	18.32	38.47	50.85
	4	0.6	0.05	29.24	66.48	89.33
16	1	0.3	0.05	3.73	10.15	11.69
	2	0.3	0.05	9.86	17.07	19.03
	4	0.3	0.05	26.03	28.71	30.98
	6	0.3	0.05	45.92	38.92	41.20
3	4	0.3	0.05	3.05	2.33	3.45
15	4	0.3	0.05	23.96	26.06	28.47

## Data Availability

The original contributions presented in this study are included in the article/Supplementary Material. Further inquiries can be directed to the corresponding author.

## Supplementary Materials

The following supporting information can be downloaded at: https://www.mdpi.com/article/10.3390/ma18194535/s1, Figure S1: Hydrogen leakage distribution contours under different leakage orifice diameters. Figure S2: Hydrogen distribution contours under different leakage orifice orientations. Figure S3: Hydrogen leakage distribution contours under different pipeline pressures. Figure S4: Hydrogen leakage distribution contours under different pipeline temperatures. Figure S5: Hydrogen leakage distribution contours under different soil porosities. Figure S6: Hydrogen leakage distribution contours under different soil particle diameters. Figure S7: Hydrogen leakage distribution contours under different soil temperatures. Figure S8: Hydrogen leakage distribution contours under different burial depths.

## Nomenclature

The following abbreviations are used in this manuscript:

a	Substance-specific van der Waals parameters (Pa·m^6^·mol^−2^)
A	Cross-sectional area of orifice (mm^2^)
b	Substance-specific van der Waals parameters (m^3^·mol^−1^)
C_O_	Courant number (-)
C_2_	Soil inertial resistance coefficient (m^−1^)
C	Scaling constant (-)
C_a_	Capillary numbers (-)
c_p_	Specific heat at constant pressure (m^2^/(s^2^∙K))
d_0_	Leakage orifice diameter (mm)
d_s_	Soil particle diameter (mm)
D_0_	Free-space diffusion coefficient (m^2^/s)
D_eff_	Effective diffusion coefficient (m^2^/s)
E	Total energy of the fluid element (J)
f	body force per unit mass (m/s^2^)
G_r_	Grashof Number (-)
G_b_, G_ωb_	Buoyancy term (m^2^/s^3^, s^−2^)
G_k_	Turbulent kinetic energy production due to the mean velocity gradient (m^2^/s^3^)
G_ω_	Generation of ω (s^−2^)
J_i_	Turbulent diffusion rate of the i-th species (m^2^/s)
k_eff_	Effective thermal conductivity (W/(m·K))
Kn	Knudsen number (-)
L	Pipeline burial depth (m)
L_c_	Characteristic length scale (m)
P_rt_	Turbulent Prandtl number (-)
p	Pressure of the hydrogen pipeline (MPa)
P_e_	Péclet number (-)
P_c_	Capillary threshold pressure (Pa)
Q	Mass flow rate (kg/s)
r_eff_	Effective pore radius (mm)
Re	Reynolds number (-)
S_k_	User-defined source item (m^2^/s^3^)
S_ω_	User-defined source item (s^−2^)
T_1_	Temperature of the hydrogen pipeline (K)
T_0_	Temperature of the soil (K)
T	Temperature (K)
(T_ij_)_eff_	Effective deviatoric stress tensor (Pa)
u_i_	Velocity in the X, Y and Z directions (m/s)
ν	Velocity vector (m/s)
ν_c_	Characteristic flow velocity (m/s)
X	Coordinates in horizontal (mm)
Y_k_	Dissipation of k due to turbulence (m^2^/s^3^)
Y_ω_	Dissipation of ω due to turbulence (s^−2^)
Y_i_	Mass fraction of the i-th species (-)
Y	Coordinate in the direction of pipe length (mm)
α	Viscous resistance coefficient (m^−2^)
α_c_	Exponents for orifice diameter (-)
α(T)	Temperature-dependent dimensionless correction factor (-)
β	Exponents for pressure (-)
Γ*_k_*	Effective diffusivity of k (m^2^/s^3^)
Γ*_ω_*	Effective diffusivity of ω (m^2^/s)
ϒ	Exponents for porosity (-)
ΔP	Pressure drop (Pa)
ΔC	Concentration gradients (-)
δ	Exponents for particle diameter (-)
θ	Orifice orientation (-)
μ	Dynamic viscosity (Pa·s)
μ_t_	Turbulent viscosity (Pa·s)
ρ	Fluid density (kg/m^3^)
τ	Soil tortuosity factor (-)
υ	Molar volume (m^3^·mol^−1^)
φ	Soil porosity (-)
χH_2_	Hydrogen Mole concentration (-)
**Abbreviate**	
LEL	lower explosive limit
MAE	Mean absolute error
RSS	Residual sum of squares
SQP	Sequential quadratic programming
UEL	Upper explosion limit
PQA	Probabilistic risk assessment
QRA	Quantitative risk assessment
UHS	Underground hydrogen storage
THMC	Coupling thermo-hydro-mechanical-chemical
