# Supplementary material for: Numerical Investigation of Hydrogen Leakage Quantification and Dispersion Characteristics in Buried Pipelines
Source: Materials (Basel). 2025 Sep 29;18(19):4535. doi: 10.3390/ma18194535 (PMC12526168; doi:10.3390/ma18194535)
Supplement: Supplementary file 1 [file materials-18-04535-s001.zip › materials-3861107-supplementary.pdf]

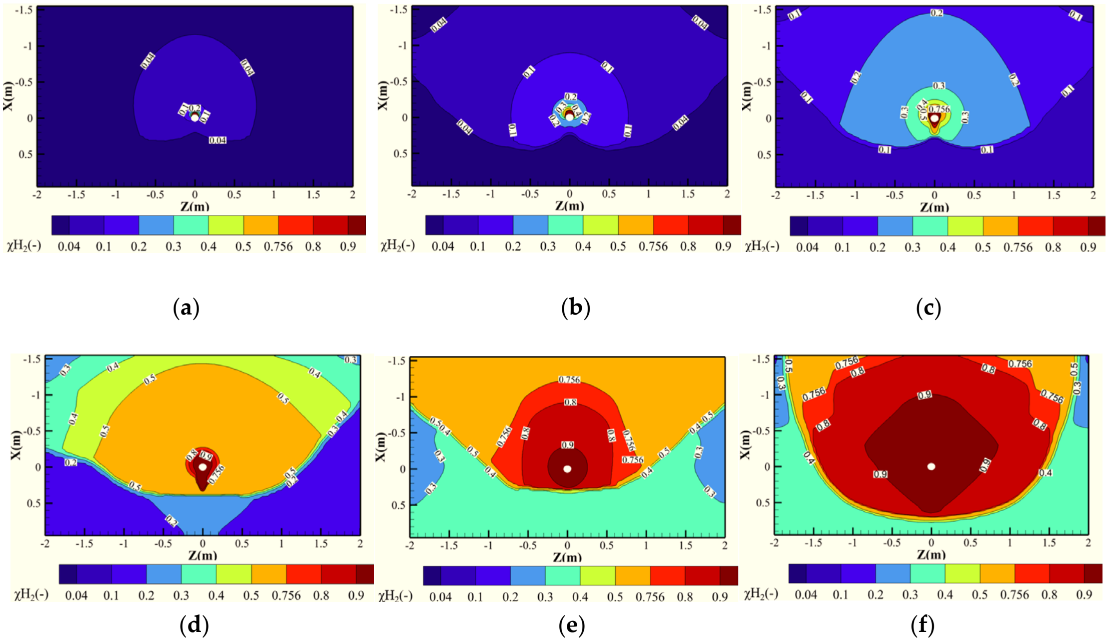

Figure S1 Hydrogen leakage distribution contours under different leakage orifice diameters.

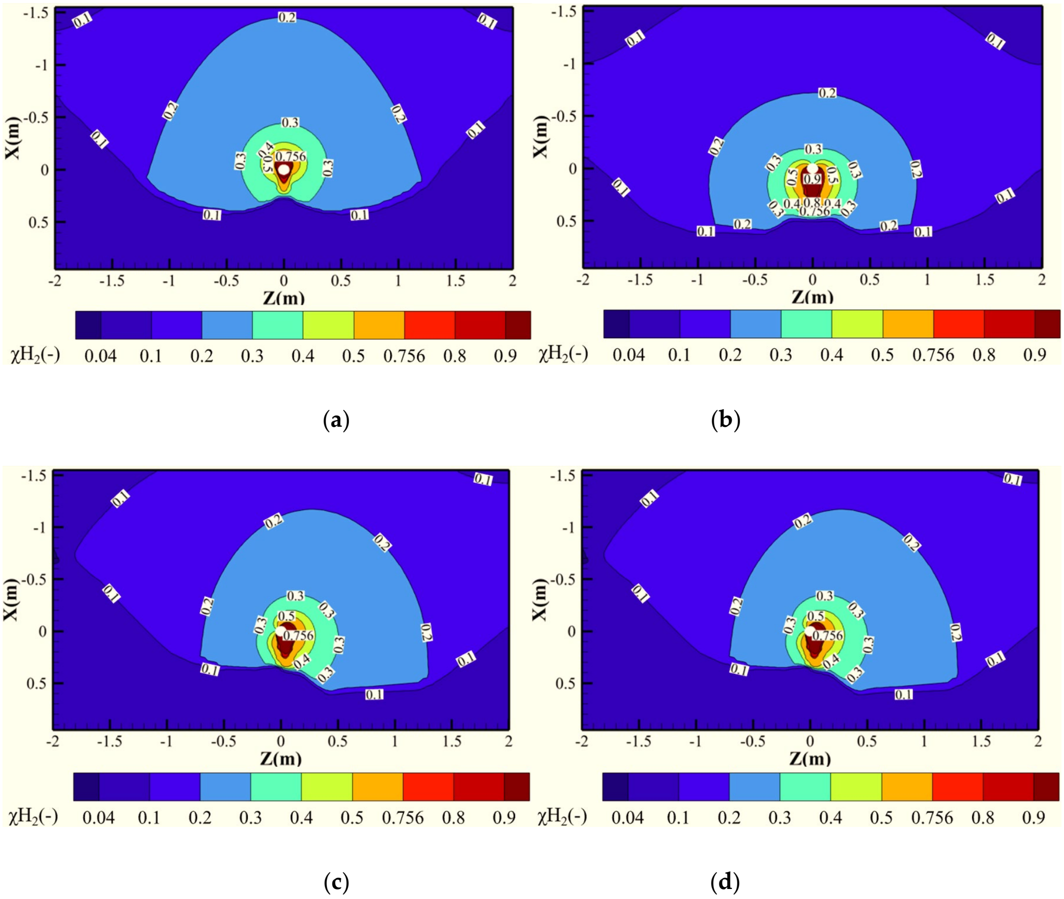

Figure S2 Hydrogen distribution contours under different leakage orifice orientations.

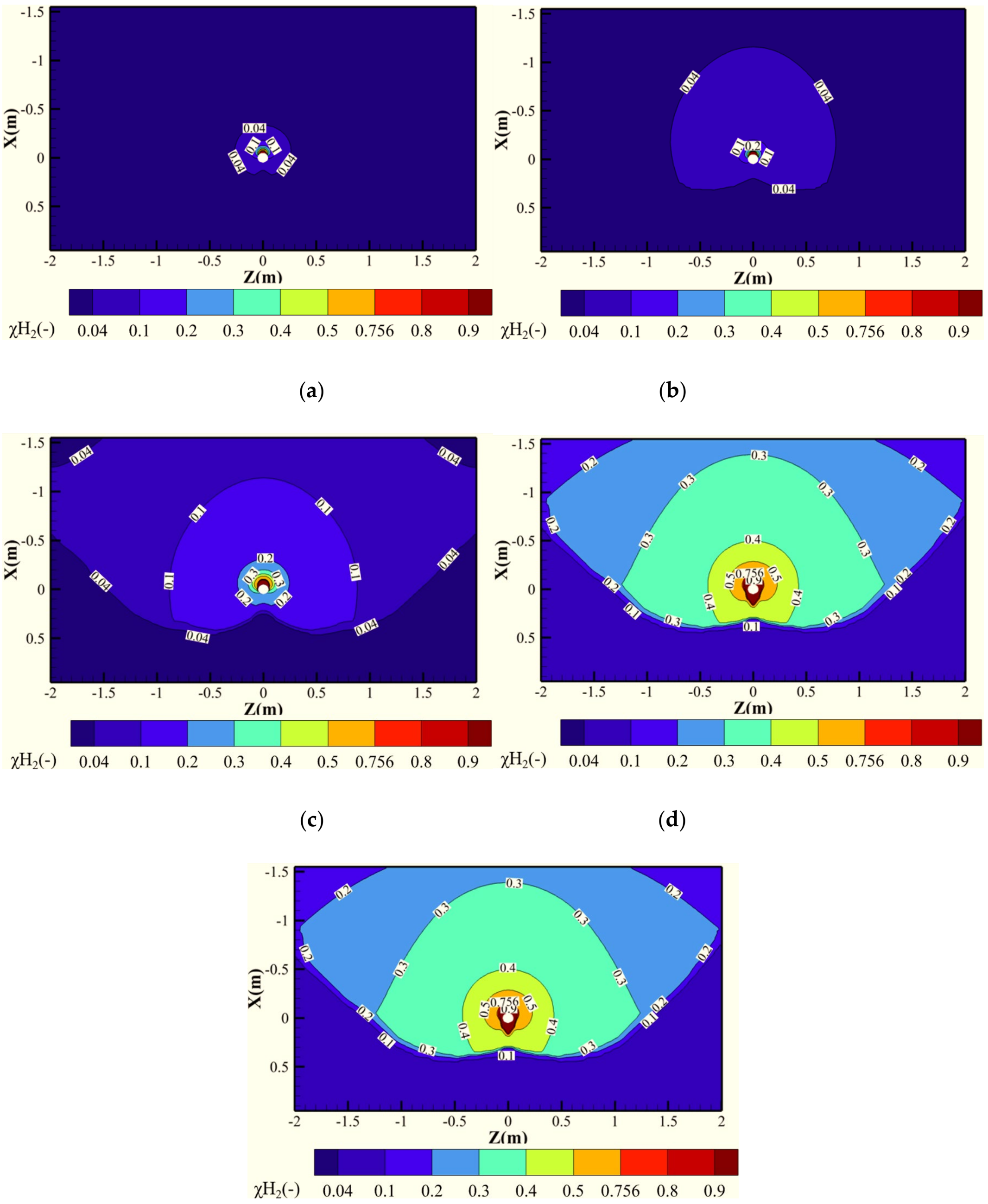

(e)

Figure S3 Hydrogen leakage distribution contours under different pipeline pressures.

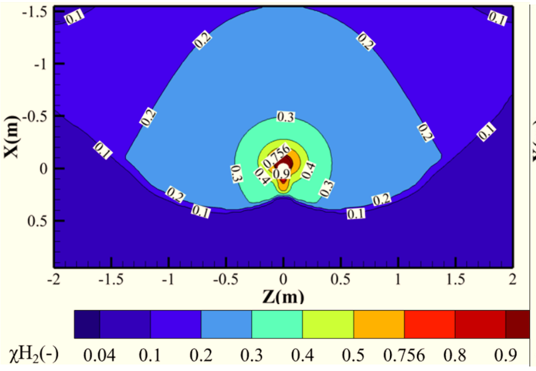

(**a**)

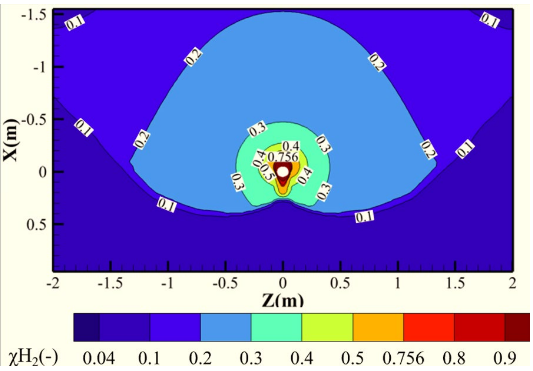

(**b**)

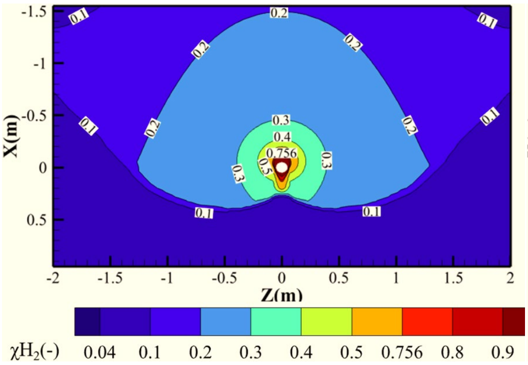

(**c**)

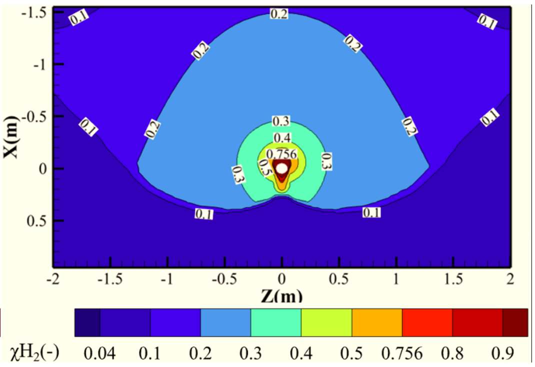

(**d**)

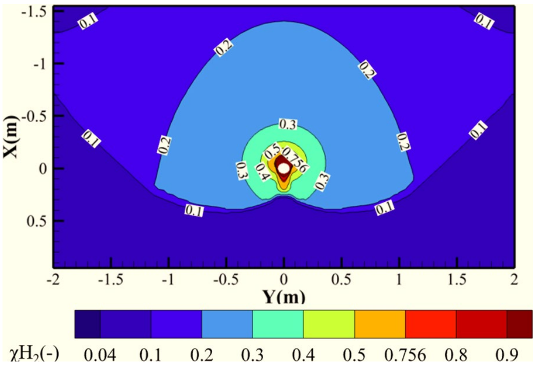

(**e**)

Figure S4 Hydrogen leakage distribution contours under different pipeline temperatures.

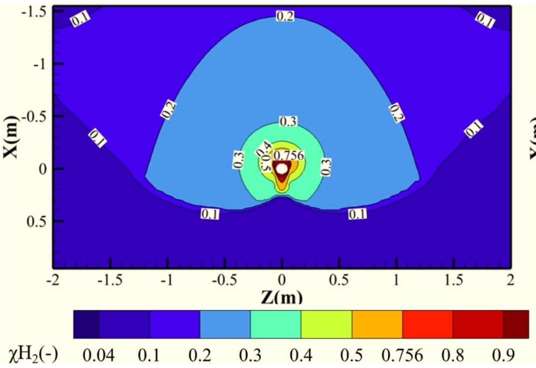

(**a**)

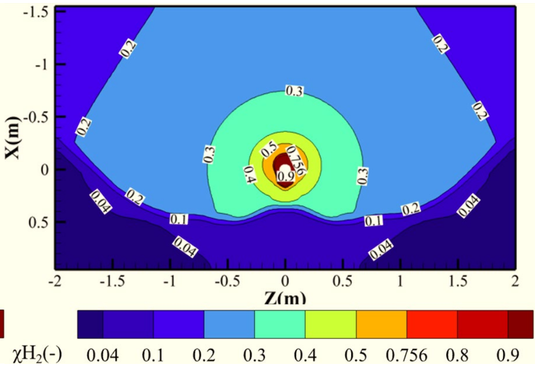

(**b**)

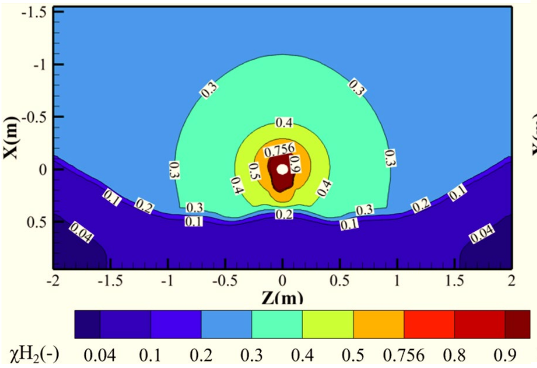

(**c**)

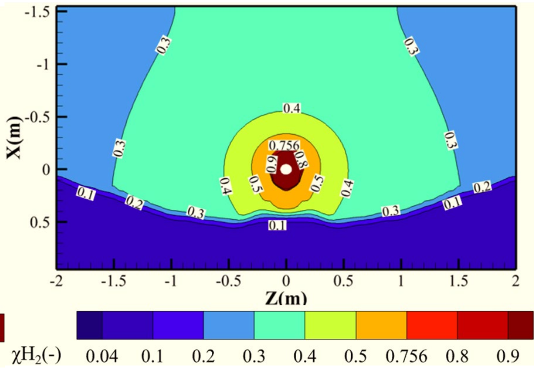

(**d**)

Figure S5 Hydrogen leakage distribution contours under different soil porosities.

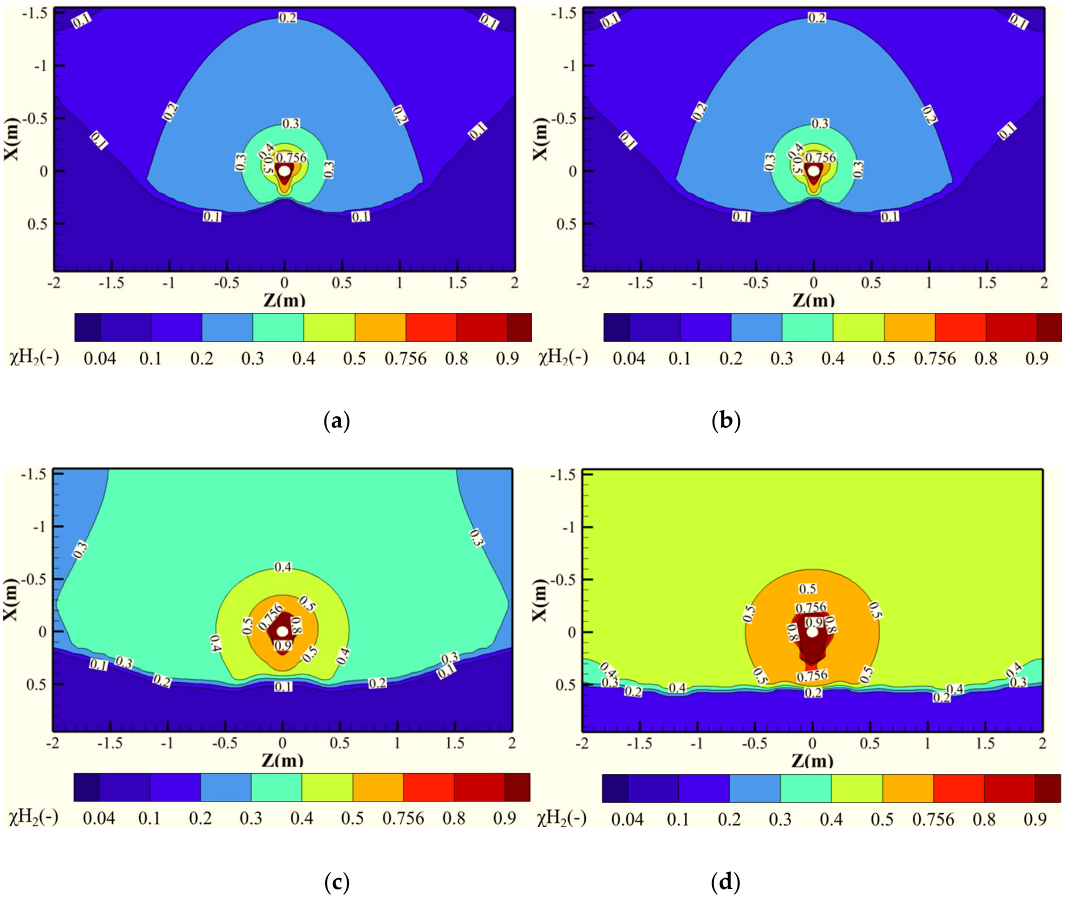

Figure S6 Hydrogen leakage distribution contours under different soil particle diameters.

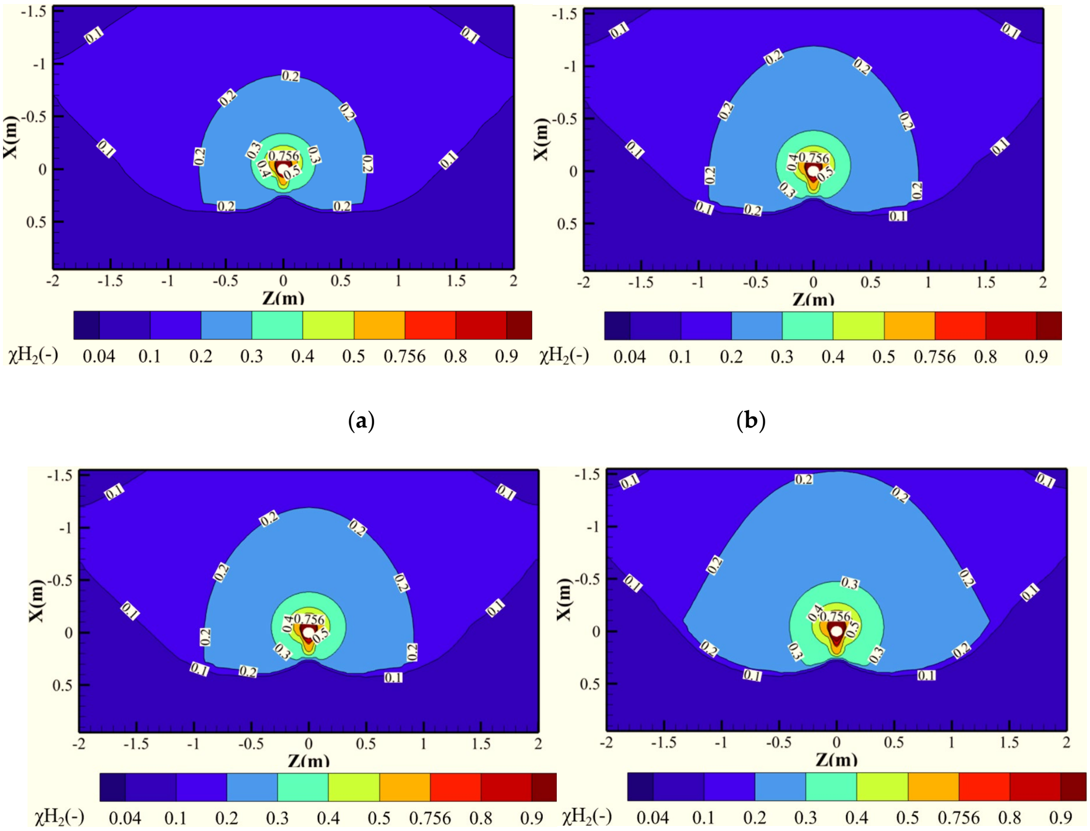

(**a**) (**b**)

(**c**) (**d**)

Figure S7 Hydrogen leakage distribution contours under different soil temperatures.

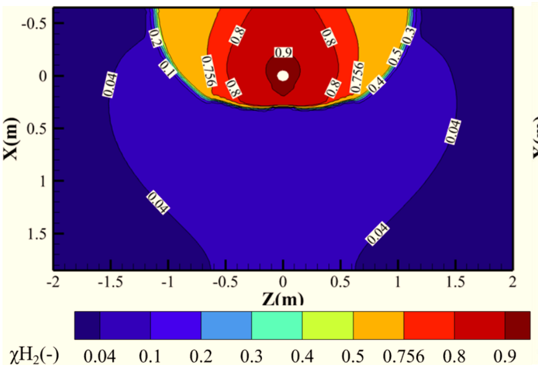

(**a**)

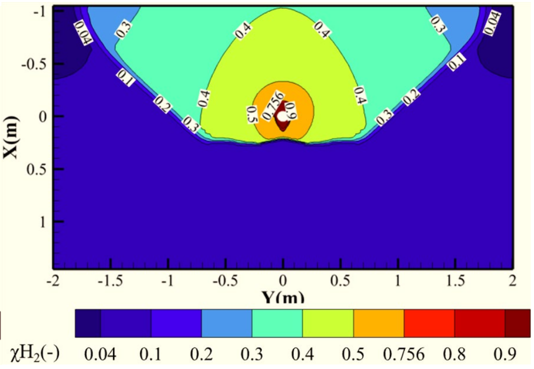

(**b**)

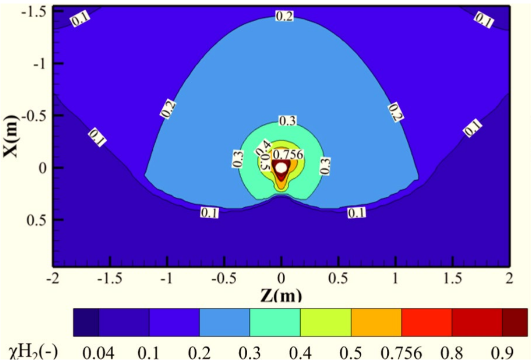

(**c**)

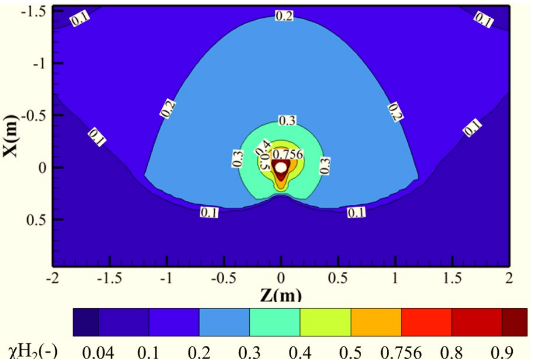

(**d**)

Figure S8 Hydrogen leakage distribution contours under different burial depths.
